# 1,8‐Diazabicyclo[5.4.0]undec‐7‐ene as Cyclic Ether Electrolyte Polymerization Inhibition for Wide‐Temperature‐Range High‐Rate Lithium‐ion Batteries

**DOI:** 10.1002/advs.202409259

**Published:** 2024-11-08

**Authors:** Hui Tian, Zixin Hong, Zhenhan Fang, Yufeng Luo, Hengcai Wu, Fei Zhao, Qunqing Li, Shoushan Fan, Jiaping Wang

**Affiliations:** ^1^ Department of Physics and Tsinghua‐Foxconn Nanotechnology Research Center Tsinghua University Beijing 100084 China; ^2^ Institute of Textiles and Clothing Hong Kong Polytechnic University Hong Kong SAR 99077 China; ^3^ Frontier Science Center for Quantum Information Beijing 100084 China

**Keywords:** cyclic ether‐based electrolytes, high rates, lithium‐ion batteries, polymerization inhibitions, wide temperatures

## Abstract

1,3‐Dioxolane (DOL), with its broad liquid phase temperature window and low Li^+^‐solvent binding energy, stands out as an ideal solvent candidate for the wide‐temperature and high‐rate electrolytes. Unfortunately, DOL is susceptible to undergo ring‐opening polymerization under common lithium salts, which markedly retards the reaction kinetics. This work introduces the organic basic additive 1,8‐Diazabicyclo[5.4.0]undec‐7‐ene (DBU) to effectively suppress the polymerization, thus achieving compatibility between LiFSI, LiDFOB lithium salts, and DOL. Furthermore, density functional theory (DFT) calculations are utilized to elucidate the underlying mechanisms of DOL polymerization and to clarify how DBU inhibits its polymerization. The resulting electrolyte, devoid of polymer chain formation, forms a weak solvation structure rich in anions, which demonstrates rapid ion transport kinetics in the bulk electrolyte and excellent electrochemical stability at the electrolyte–electrode interfaces (EEIs) simultaneously. When applied to the LiFePO_4_||graphite full cell, it exhibits exceptional wide‐temperature and high‐rate performance, with specific capacities reaching 101.2 mAh g ^−1^ at room temperature (20 C), 36.9 mAh g^−1^ at −40 °C (0.5 C), and 118.0 mAh g^−1^ at 60 °C (20 C). This study significantly guides the development of wide‐temperature, high‐rate electrolytes.

## Graphical abstract

By incorporating a trace amount of the additive 1,8‐Diazabicyclo[5.4.0]undec‐7‐ene (DBU), a complete inhibition of 1,3‐dioxolane (DOL) polymerization in the dual‐lithium salt system comprising LiFSI and LiDFOB is achieved. This strategy maintains a stable weak solvation structure of the electrolyte over a wide temperature range, enabling the stable operation of LiFePO_4_||graphite lithium‐ion batteries.

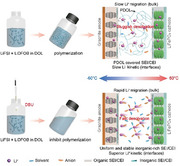

## 1 Introduction

Lithium‐ion batteries (LIBs) are increasingly used as the dominant power source in electric vehicles (EVs).^[^ ^1^, ^2^ ^]^ However, the rapid degradation of electrochemical performance under extreme temperature conditions and the long charging time from empty to full states are the two main bottlenecks restricting the further development of EVs. To address these issues, there is an urgent need to develop LIBs with a wide operating temperature range and high rate capability, enabling EVs to adapt to diverse regions and climates and achieve “extreme fast charging” (XFC),^[^ ^3^ ^]^ which would greatly enhance the practicality and market competitiveness of EVs.^[^ ^4^, ^5^ ^]^ Currently, most commercial LIBs have a narrow operating temperature range (typically only capable of discharging from −20 to 55 °C and recharging from 0 to 45 °C), and the reversible specific capacity usually decreases rapidly at high charge/discharge rates.^[^ ^6^, ^7^ ^]^ These limitations are primarily attributed to the sluggish kinetics of lithium‐ion (Li^+^) transport within the electrolyte bulk phase and across the electrolyte–electrode interfaces (EEIs).^[^ ^8^, ^9^ ^]^ Elucidating and manipulating the microscopic solvation structure of Li^+^ in electrolytes is crucial for influencing the macroscopic properties of electrolytes and EEIs, and it represents a focal point in the ongoing research on LIBs. Chen et al.^[^ ^10^ ^]^ revealed that the final solvation structures of Li^+^ in electrolytes are determined by the competition between Li^+^‐solvent (ion‐dipole) interactions and Li^+^‐anion (ion‐ion) interactions. Subsequently, Chen et al.^[^ ^11^ ^]^ introduced an electron‐donation modulation (EDM) rule for creating LiNO_3_‐based electrolytes by utilizing low donor‐number solvents (LDNSs) such as propylene carbonate (PC) to regulate the solvation structure, thereby enhancing electrolyte stability. Luo et al.^[^ ^12^ ^]^ employed the weak‐solvation isobutyronitrile as cosolvent to facilitate the operation of LIBs at ultra‐low temperatures. Recent studies have increasingly demonstrated that the weak solvation structures of the electrolytes facilitate the rapid switching and migration of Li^+^ between different solvation structures in the electrolyte bulk phases, as well as the rapid desolvation process at the EEIs, significantly accelerating the ion transport efficiency.^[^ ^13^, ^14^ ^]^ Moreover, weak solvation structures often contain a large number of anions, leading to the formation of solid electrolyte interphase (SEI) and cathode electrolyte interphase (CEI) rich in inorganic components with low electronic conductivity, high ionic conductivity, and mechanical strength. This accelerates the transport of Li^+^ at the EEIs while enhancing interface stability.^[^ ^15^, ^16^, ^17^, ^18^ ^]^ Therefore, designing electrolytes with weak solvation structures can be a key breakthrough for effectively improving the high‐rate charge and discharge performance of LIBs over a wide temperature range.^[^ ^8^, ^15^, ^19^ ^]^

**Figure 1:**
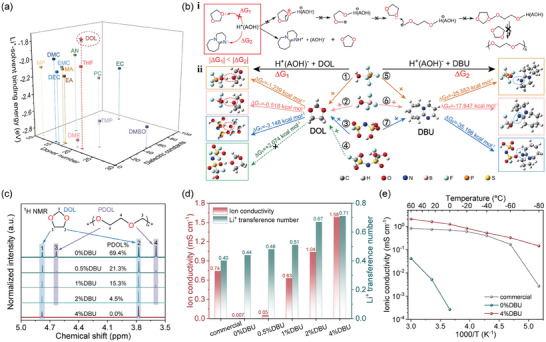
The inhibition mechanism of DBU on DOL polymerization and electrolyte property characterization. a) The solvent diagram of DN, dielectric constant, and Li^+^‐solvent binding energy; b) i: Schematic illustration of the mechanism by which DBU inhibits DOL polymerization (“A” represents free radicals derived from lithium salts), ii: The reaction processes of H^+^A(OH)^−^ derived from 1)LiPF_6_, 2)LiBF_4_ (and LiDFOB), 3) LiFSI, 4) LiTFSI with DOL, and the reaction processes of H^+^A(OH)^−^ derived from 5)LiPF_6_, 6) LiBF_4_ (and LiDFOB), 7) LiFSI with DBU. c) ^1^H NMR spectra of commercial electrolyte and DOL‐based electrolytes with different DBU contents after one week of standing; d) Ionic conductivity and Li^+^ transference number of commercial electrolyte and DOL‐based electrolytes with different DBU contents; e) The relationship between ionic conductivity and temperature for the commercial, 0%DBU, and 4%DBU electrolytes.

Compared to various commercial solvents such as ethylene carbonate (EC) and PC, 1,3‐dioxolane (DOL) offers notable advantages, including a broad liquid‐phase temperature range (melting point of −95 °C and boiling point of 74 °C), a moderate donor number (DN) of 20, and a low dielectric constant of 7.3. These properties ensure efficient lithium salt dissociation across a wide temperature range while minimizing the Li^+^‐solvent binding energy. Density functional theory (DFT) calculations have confirmed that DOL possesses a relatively low Li^+^‐solvent binding energy (−1.713 eV), making it an effective solvent for constructing electrolytes with weak solvation structures. Considering these advantages, DOL emerges as an ideal candidate for wide‐temperature, fast‐charging electrolytes (**Figure** **1**a; Table S1, Supporting Information).^[^ ^20^, ^21^, ^22^ ^]^ Currently, DOL‐based electrolytes using lithium bis(trifluoromethanesulfonyl)imide (LiTFSI) as the salt have been extensively investigated. However, Xia et al.^[^ ^23^ ^]^ found that substituting the larger TFSI^−^ with the smaller anion (bis(fluorosulfonyl)imide anion, FSI^−^) could effectively reduce the steric hindrance of the anions, promoting their involvement in the Li^+^ solvation structure and increasing the proportion of aggregates (AGG), thereby facilitating the formation of weak solvation structures. Unfortunately, aside from a few salts like LiTFSI, common lithium salts such as lithium bis(fluorosulfonyl)imide (LiFSI), LiPF_6_, LiBF_4_, and lithium difluoro(oxalate)borate (LiDFOB) pose a risk of inducing in situ polymerization of the cyclic ether DOL into poly(1,3‐dioxolane) (PDOL).^[^ ^10^, ^24^, ^25^ ^]^ This gelation phenomenon severely hampers the ionic transport kinetics in the bulk electrolyte and at the EEIs, accompanied by a series of deteriorations in the electrochemical performance, particularly at low temperatures and high charge–discharge rates.^[^ ^26^ ^]^ Consequently, resolving the DOL polymerization issue is essential to expand the compatibility of DOL with other lithium salts and realize weak solvation structures enriched with anions, thereby fully harnessing the potential of DOL in wide‐temperature LIBs.

Current research has elucidated the polymerization mechanisms of DOL induced by lithium salts such as LiPF_6_,^[^ ^27^ ^]^ LiBF_4_,^[^ ^28^ ^]^ LiDFOB,^[^ ^29^ ^]^ and LiFSI.^[^ ^30^ ^]^ These reactions are commonly initiated by hydrated protonic acids (H^+^A(OH)^−^), which are formed from trace amounts of H_2_O and Lewis acidic radicals (e.g., PF_5_, BF_3_, etc., denoted as “A”) derived from the lithium salts. The proton in H^+^A(OH)^−^ tends to attack the lone pair electrons on the oxygen atoms in DOL, thereby disrupting the epoxy bond and initiating the ring‐opening polymerization of DOL. The DOL monomer undergoes rapid protonation to produce secondary oxygen ions, which are repeatedly inserted into the reaction sequence, leading to the growth of the DOL polymer into PDOL. Among these reactions, H^+^A(OH)^−^ is the critical factor that initiates the ring‐opening polymerization of DOL. Therefore, to effectively prevent DOL polymerization, “acid scavengers” are introduced to block the attack of H^+^A(OH)^−^ on DOL monomers and fundamentally suppress the ring‐opening process. Table S2 (Supporting Information) compares various common organic bases, demonstrating that 1,8‐Diazabicyclo[5.4.0]undec‐7‐ene (DBU) possessed potent basicity (*pK*_a_ = 11.5 in water and 23.9 in acetonitrile) and minimal electrostatic potential (ESP_min_, −45.475 kcal mol^−1^), which facilitated its reaction with protonic acids (Figure S6, Supporting Information illustrated the ESP distribution of DBU). Additionally, other comprehensive benefits including superior nucleophilicity and a broad temperature range (−70 to 275 °C) collectively positioned DBU as an optimal additive selection.^[^ ^31^, ^32^ ^]^

Herein, we introduced DBU as an additive into DOL‐based electrolyte systems. Upon the addition of this organic basic additive, the protons in H^+^A(OH)^−^ were more inclined to bind with the lone pair of electrons on the imine nitrogen atom of DBU, rather than attacking the lone pair of electrons on the oxygen atom of the DOL ether bond. This effectively inhibited the ring‐opening reaction of DOL, thus completely blocking the subsequent monomer polymerization reaction (Figure 1b(i)). By combining DBU with a dual‐salt system of LiFSI and LiDFOB, an electrolyte (0.8 m LiFSI + 0.2 m LiDFOB in DOL with 4 vol% DBU, denoted as “4%DBU”) with rapid Li^+^ transport kinetics over a wide temperature range was obtained. Compared to the commercial EC‐based electrolyte (denoted as “commercial”) and the DOL‐based electrolyte without DBU addition after polymerization (denoted as “0%DBU”), graphite and LiFePO_4_ (LFP) electrodes exhibited excellent wide‐temperature electrochemical performance with the 4%DBU electrolyte. Reversible charge and discharge at extremely low temperatures of −70 °C for graphite anodes and −50 °C for LFP cathodes were achieved. At both room temperature and high temperatures of 60 °C, ultra‐high rate capabilities of 50 C for graphite anodes (> 150 mAh g^−1^) and 20 C for LFP cathodes (> 100 mAh g^−1^) were attained. Full cells assembled with the 4%DBU electrolyte also demonstrated superior electrochemical performance from −60 to 60 °C, achieving high‐rate performance at 20 C with specific capacities of 101.2 mAh g^−1^ at 25 °C and 118.0 mAh g^−1^ at 60 °C, as well as 36.9 mAh g^−1^ at 0.5 C at −40 °C. The addition of a small amount of DBU effectively broadened the lithium salt compatibility, wide‐temperature adaptability, and high‐rate performance of DOL‐based electrolytes, economically paving the way for the development of commercial wide‐temperature and high‐rate LIBs.

## 2 Results and Discussion

### 2.1 Mechanism of DBU inhibition of DOL Polymerization and Fundamental Electrolyte Characterization

To elucidate the mechanism by which DBU inhibits DOL polymerization, DFT calculations were conducted to determine the Gibbs free energy changes (∆*G*_1_ and ∆*G*_2_) for the reactions of H^+^A(OH)^−^ with DOL and DBU, in order to assess the spontaneity and relative difficulty of the reactions. The ∆*G*_1_ values for the LiPF_6_, LiBF_4_ (and LiDFOB), and LiFSI systems were found to be −1.239, −0.518, and −3.148 kcal mol^−1^, while the ∆*G*_2_ values were −25.553, −17.647, and −35.198 kcal mol^−1^. It is evident that the absolute values of ∆*G*_2_ are significantly greater than those of ∆*G*_1_, indicating that the protons in H^+^A(OH)^−^ are more likely to attack the nitrogen atom in DBU rather than the oxygen atom in DOL, thereby effectively blocking the ring‐opening polymerization reaction of DOL. Moreover, these computational results also explain why LiTFSI does not induce DOL polymerization (∆*G*_1_ > 0) (Figure 1b).

Nuclear magnetic resonance (^1^H NMR) spectroscopy was employed to assess the occurrence of DOL polymerization and to quantify the degree of polymerization. Specifically, chemical shifts at 3.78 ppm (─OCH_2_CH_2_O─) and 4.78 ppm (─OCH_2_O─) were attributed to DOL, while peaks at 4.63 ppm and 3.60 ppm corresponded to PDOL. The degree of polymerization can be estimated by integrating the ^1^H NMR signals of DOL and PDOL.^[^ ^33^ ^]^ Initially, ^1^H NMR spectra of 0.8 m LiFSI + 0.2 m LiDFOB in DOL without DBU were recorded after the solution was allowed to stand for 0 hours, 2 days, 1 week, and 2 weeks (Figure S7, Supporting Information). The results showed that the electrolyte polymerization reached saturation at 1 week (≈70%), establishing 1 week as the standard condition for subsequent experiments. Then DBU was introduced at volume fractions of 0%, 0.5%, 1%, 2%, and 4%, with NMR spectra analysis indicating that only a small concentration of 4% DBU was sufficient to completely inhibit PDOL formation (Figure 1c).

The 4%DBU electrolyte exhibited enhanced wettability on the electrode and separator surfaces compared to the commercial electrolyte, as indicated by lower viscosity (2.95 mPa·s vs 4.41 mPa·s) and contact angle (21.7° vs 40.6°) measurements at room temperature (Figure S8, Supporting Information). Figure S9 (Supporting Information) presents the differential scanning calorimetry (DSC) and thermogravimetric (TG) thermal profiles for three electrolytes. The 4%DBU electrolyte exhibited a low freezing point below −120 °C. The 0%DBU electrolyte underwent a glass transition at ≈−59 °C, while the commercial electrolyte solidified between −30 and −20 °C. To further investigate the impact of DBU inhibition on DOL polymerization, the ionic conductivity and Li^+^ transference number of a series of electrolytes with varying DBU concentrations were measured at room temperature. As the polymerization degree of DOL decreased from ≈70% to 0%, the ionic conductivity of the electrolyte increased from 0.007 to 1.58 mS cm^−1^, and the Li^+^ transference number also rose from 0.44 to 0.71 (Figure 1d, Figures S10 and S11, Supporting Information). The ionic conductivities of the commercial, 0%DBU, and 4%DBU electrolytes over a temperature range of −80 °C to 60 °C were also tested (Figure 1e). The commercial electrolyte displayed a more rapid decay in ionic conductivity below −40 °C, which was attributed to the higher melting points of EC (36.4 °C) and DMC (4 °C).^[^ ^21^ ^]^ The 0%DBU electrolyte, characterized by extensive polymeric segments, exhibited significantly sluggish transport efficiency due to Li^+^ migration through coordination and un‐coordination with electron donor sites in molecular chains,^[^ ^34^ ^]^ resulting in ionic conductivity three orders of magnitude lower than the commercial electrolyte at 0 °C. In comparison, the 4%DBU electrolyte showcased superior ionic conductivity from −80 °C to 60 °C, maintaining 0.14 mS cm^−1^ even at −80 °C. These findings indicate that the inhibition of DOL polymerization by DBU accelerates the ion transport kinetics of the electrolyte bulk phase, thereby reducing concentration polarization under high‐current charging and discharging conditions.

### 2.2 Characterization of the Electrolyte–Electrode Interfaces

In addition to accelerating the ion diffusion and migration kinetics within the electrolyte bulk phase over a wide temperature range, the inhibition of DOL polymerization by DBU can also exert a significant influence on the electrolyte‐graphite/LFP electrode interfaces.

**Figure 2:**
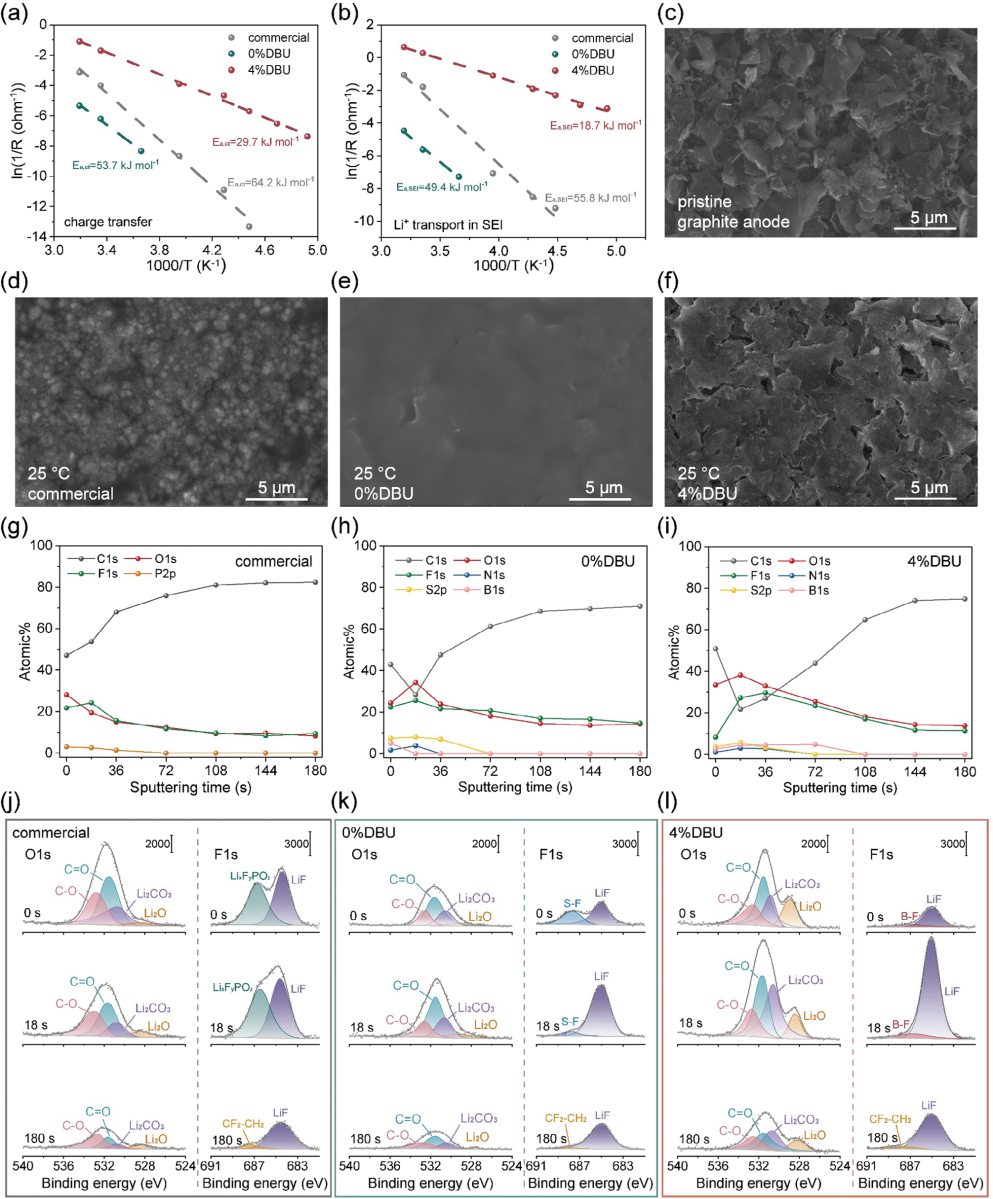
Characterization of graphite electrode surfaces in the three electrolytes. Activation energy for a) the charge transfer process and b) Li^+^ transport through SEI; SEM images of the c) pristine graphite electrode surface and the graphite electrode surfaces following electrochemical testing at 25 °C in the d) commercial, e) 0%DBU, and f) 4%DBU electrolytes (Figure S14, Supporting Information presents SEM images after testing at −40 °C and 60 °C); XPS general spectra of the SEI on graphite anodes after Li^+^ deintercalation at room temperature for the g) commercial, h) 0%DBU, and i) 4%DBU electrolytes; XPS O1s and F1s spectra of the SEI on graphite anodes after Li^+^ deintercalation at room temperature with the j) commercial, k) 0%DBU, and l) 4%DBU electrolytes (Figures S15 for C1s spectra and S16 for N1s spectra, Supporting Information).

To compare the kinetic rates of Li^+^ transport at the electrolyte‐graphite anode interfaces under different electrolyte systems, electrochemical impedance spectroscopy (EIS) tests were conducted on graphite||Li half cells using the commercial, 0%DBU, and 4%DBU electrolytes at various temperatures (Figure S12, Supporting Information). The data were fitted using the equivalent circuit depicted in Figure S13, Supporting Information. By fitting the charge transfer resistance (*R*_ct_) and the transport resistance of Li^+^ in the SEI (*R*_SEI_) values for each electrolyte at different temperatures according to the Arrhenius equation, the activation energies for the charge transfer process (*E*_a,ct_) and Li^+^ transport through the SEI (*E*_a,SEI_) were determined (**Figure** **2**a,b).^[^ ^35^ ^]^ The *E*_a,ct_ and *E*_a,SEI_ for the 4%DBU electrolyte at the graphite interface were 29.7 and 18.7 kJ mol^−1^, which were significantly lower than those of the commercial electrolyte (64.2 and 55.8 kJ mol^−1^) and the 0%DBU electrolyte (53.7 and 49.4 kJ mol^−1^). These results indicate that the non‐polymerizing DOL‐based electrolyte enhances the interfacial kinetics and reduces the polarization of the graphite anode effectively.

To further investigate the underlying reasons, scanning electron microscopy (SEM) was employed to characterize the surface morphologies of the pristine graphite electrode (Figure 2c) and the graphite electrodes retrieved after the rate testing at −40, 25, and 60 °C (Figure 2d–f; Figure S14, Supporting Information). The surfaces of the graphite electrodes in the 0%DBU electrolyte system were completely covered by the low ionic conductivity PDOL film, which is the primary reason for the slow interfacial transport kinetics of Li^+^ (Figure 2e; Figures S14b,e, Supporting Information). In the commercial electrolyte system, graphite surfaces were rough and uneven at 25 and 60 °C. At −40 °C, minimal SEI formation was observed due to the negligible Li^+^ conduction and resulting lack of electrochemical reactions (Figure 2d; Figures S14a,d, Supporting Information). In contrast, under the 4%DBU electrolyte system, the graphite surfaces were free of any polymer coverage, and a dense, smooth, and uniform SEI was formed at −40, 25, and 60 °C (Figure 2f; Figures S14c,f, Supporting Information), which facilitated reversible charge‐discharge cycling of the graphite electrode at wide temperatures and high rates without side reactions and with reduced polarization.

The chemical composition and structural characteristics of the graphite surfaces after delithiation at room temperature in different electrolyte systems were further analyzed by Ar^+^ sputtering X‐ray photoelectron spectroscopy (XPS). The signals were collected at Ar^+^ etching times of 18, 36, 72, 108, 144, and 180 seconds (Figure 2g–i shows the general spectra, Figure 2j–l shows the O1s and F1s spectra, Figure S15, Supporting Information shows the C1s spectra, and Figure S16, Supporting Information shows the N1s spectra). For the 0%DBU electrolyte system, XPS spectra were obtained after removing most surface polymers during cleaning (Figure S17, Supporting Information shows the general spectrum obtained from the electrode with the uncleaned surface polymer). The SEI formed by the commercial electrolyte exhibited an increasing atomic percentage of C elements with Ar^+^ etching time, while the percentages of O, F, and P elements generally decreased, indicating the formation of the SEI rich in organic materials. After etching for 108 seconds, the elemental ratios stabilized, with the carbon content reaching its highest point (≈80%), indicating that Ar^+^ etching has approached the anode surface (Figure 2g). The SEI formed by the 4%DBU electrolyte showed a decreasing then increasing trend in C elemental percentage with etching time, while the percentages of O and F elements increased initially and then decreased reaching a minimum of 21.64% for C, and a maximum of 38.19% for O and 27.21% for F at 18 seconds. These results suggest a distinct layered SEI structure, with an outer layer composed of a mixture of organic and inorganic materials and an inner layer predominantly consisting of dense inorganic substances. Similarly, after 108 seconds of Ar^+^ sputtering, the elemental percentages stabilized, and the signals for B, N, and S elements disappeared, indicating proximity to the anode surface (Figure 2i). The SEI formed by the 0%DBU electrolyte showed similar trends in elemental percentage changes with the 4%DBU electrolyte, suggesting a layered structure (Figure 2h). However, the mixture of SEI and polymer electrolyte at the interface significantly retarded the kinetic process of Li^+^ transport through the SEI. Specifically, the narrow spectra analysis of C1s, O1s, and F1s showed that the commercial electrolyte produced more organic components such as C─C/C─H, C─O, C═O, and Li_x_F_y_PO_z_, primarily derived from the decomposition of EC, DMC, EMC solvent molecules and a minor contribution from LiPF_6_. The peak percentages of chemical bond signals did not change significantly with Ar^+^ etching time, indicating an intertwined and non‐layered distribution of these components, which may cause Li^+^ to encounter different energy barriers during transport through the SEI, making the transport more difficult. Additionally, at 180 seconds of Ar^+^ etching, the CF_2_‐CH_2_ peak signal in the F1s spectra originated from the PVDF binder, proving that Ar^+^ etching reached the electrode surface (Figure 2j; Figure S15a, Supporting Information). In the narrow spectra of the SEI generated by the 0%DBU electrolyte system, the peak intensities for each chemical state were generally lower than those for the 4%DBU electrolyte system. The possible reasons for this observation may include not only the less dense SEI formation but also the shielding effect of the residual low‐conductivity polymer, which may have weakened the signal intensity. The presence of the polymer is a primary factor that constrains the rapid transport of Li^+^ at the electrolyte‐graphite anode interfaces (Figure 2k; Figures S15b and S16a, Supporting Information). Compared to the other two electrolytes, the 4%DBU electrolyte could form an inorganic inner layer composed of LiF, Li_2_CO_3_, and Li_2_O, among others. LiF primarily resulted from the reduction decomposition of fluorinated anions FSI^−^ and DFOB^−^, possessing advantages such as low electron conductivity, high mechanical strength, and high interfacial energy with lithium.^[^ ^36^, ^37^ ^]^ The synergistic effect of LiF and Li_2_CO_3_ could promote the accumulation of space charge along their contact interfaces, significantly improving Li^+^ transport.^[^ ^38^ ^]^ Li_2_O contributed to reducing nucleation overpotential and lowering the surface diffusion energy barrier to Li^+^.^[^ ^39^ ^]^ The Li_3_N and Li_x_NO_y_ observed in the N1s spectra, derived from the decomposition of FSI^−^, also exhibited higher ionic conductivity.^[^ ^40^ ^]^ (Figure 2l; Figures S15c and S16b, Supporting Information) The synergistic action of various inorganic components allowed the SEI to be electronically insulating while also enabling efficient Li^+^ conduction, which helped to maintain the structural stability of the SEI on the graphite anode surface and reduce electrochemical polarization at wide temperatures and high rates.

**Figure 3:**
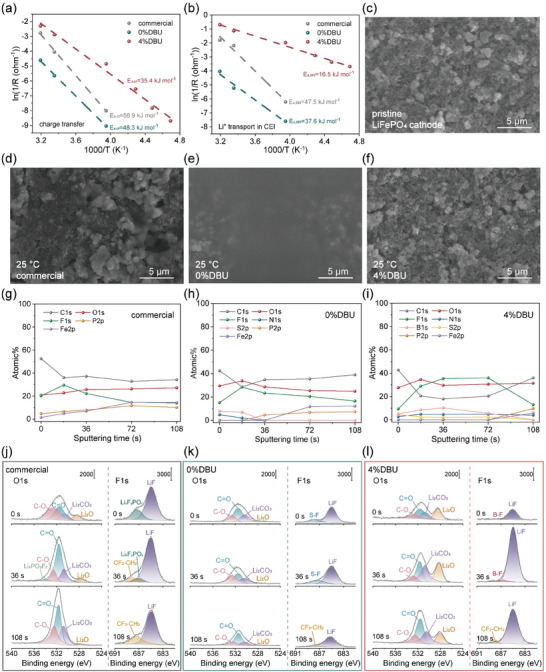
Characterization of LFP electrode surfaces in the three electrolyte systems. The activation energy for a) the charge transfer process and b) Li^+^ transport through SEI; SEM images of the c) pristine LFP electrode surface and LFP electrode surfaces following electrochemical testing at 25 °C with the d) commercial, e) 0%DBU, and f) 4%DBU electrolytes (Figure S19, Supporting Information presents SEM images after testing at −40 °C and 60 °C); XPS general spectra of the CEI on LFP cathodes after Li^+^ deintercalation at room temperature for the g) commercial, h) 0%DBU, and i) 4%DBU electrolytes; XPS O1s and F1s spectra of the CEI on LFP cathodes after Li^+^ deintercalation at room temperature with the j) commercial, k) 0%DBU, and l) 4%DBU electrolytes (Figures S20 for C1s spectra and S21 for N1s spectra, Supporting Information).

The electrolyte systems furthermore critically influenced the Li^+^ interfacial transport at the electrolyte‐LFP cathode interface, profoundly affecting battery charge and discharge kinetics.^[^ ^41^ ^]^ The *E*_a,ct_ and *E*_a,CEI_ for the 4%DBU electrolyte at the LFP interface were 35.4 and 16.5 kJ mol^−1^, which were significantly lower than those for the commercial electrolyte (56.9 and 47.5 kJ mol^−1^) and the 0%DBU electrolyte (48.3 and 37.6 kJ mol^−1^), confirming the effectiveness of the DBU‐inhibited DOL strategy for accelerating the electrolyte‐LFP interface dynamics (Figure S18, Supporting Information; **Figure** **3**a,b). Similar to the graphite surface, in the 0%DBU electrolyte system, the LFP electrode surface was completely covered by a PDOL film, and the CEI formed by the commercial electrolyte at room and high temperatures exhibited a rough, loose, and uneven morphology. In contrast, in the 4%DBU electrolyte system, a dense and uniform CEI was formed on the LFP surface at −40, 25, and 60 °C, without any coverage by low ionic conductivity polymer (Figure 3c–f; Figure S19, Supporting Information). This provided significant advantages over the other two: higher interfacial structural stability and faster Li^+^ transport kinetics across the interface within a wide temperature range. Ar^+^ sputter XPS was used to test the functional group composition and chemical evolution of the LFP electrode surfaces after delithiation at room temperature for the three electrolyte systems, with signals collected at Ar^+^ etching times of 18, 36, 72, and 108 seconds (Figure 3g–i shows the general spectra, Figure 3j–l shows the O1s and F1s spectra, Figures S20 and S21 (Supporting Information) show the C1s and N1s spectra). The spectral data for the 0%DBU electrolyte system were obtained after the removal of most of the polymer during cleaning (Figure S22, Supporting Information shows the general spectrum of the uncleaned surface polymer). Analysis revealed that the CEI formed by the commercial electrolyte system consisted of a mixture of organic components such as C─C/C─H, C─O, C═O, and Li_x_F_y_PO_z_, and a small amount of inorganic materials, without a distinct layered structure. Notably, at 0 seconds of Ar^+^ etching, the presence of P and Fe elements indicated that the CEI formed by the commercial electrolyte was not dense and failed to completely cover the surface of the active material, unable to effectively block continuous electrochemical side reactions during charge‐discharge cycles, which could potentially degrade battery performance. In contrast, the CEI formed by the 4%DBU electrolyte system had an obvious layered structure, with an outer layer composed of a mixture of organic and inorganic materials and an inner layer rich in inorganic substances such as LiF, Li_2_CO_3_, Li_2_O, Li_3_N, and Li_x_NO_y_, endowing the CEI with high mechanical strength, low electrical conductivity, and high ionic conductivity.

### 2.3 Li^+^ Solvation of Electrolytes

The above studies have experimentally confirmed that the 4%DBU electrolyte exhibited faster ion transport kinetics in both the bulk phase and EEIs, as well as forming more robust SEI and CEI, compared to the commercial and 0%DBU electrolyte systems. To delve into the intrinsic microscopic mechanisms, computational and experimental characterizations of the solvation structures of the three electrolyte systems were conducted.

**Figure 4:**
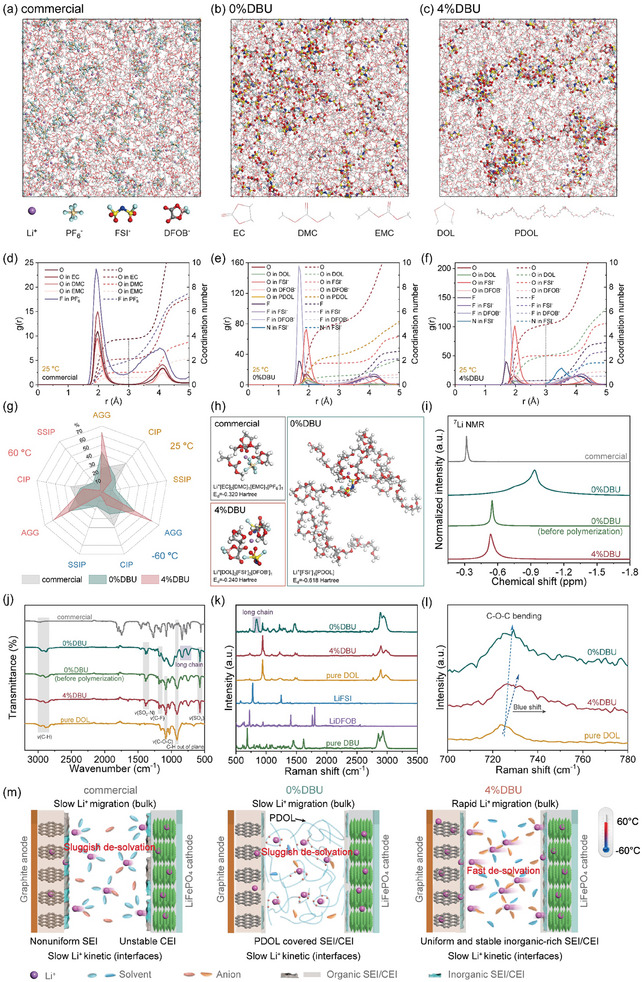
Li^+^ solvation structures in the electrolytes. MD simulation snapshots of the a) commercial, b) 0%DBU, and c) 4%DBU electrolytes at 25 °C (Figure S23, Supporting Information for snapshots at −60 °C and 60 °C); RDFs and CNs for the d) commercial, e) 0%DBU, and f) 4%DBU electrolytes at 25 °C (Figure S24, Supporting Information for RDFs and CNs at −60 °C and 60 °C); g) Radar charts of the SSIP, CIP, and AGG proportions in the three electrolytes at 25 °C, −60 °C, and 60 °C; h) representative solvation structures of the primary Li^+^ solvation shells in the three electrolytes (Figure S25, Supporting Information for other representative solvation structures); i) ^7^Li NMR of the commercial, 0%DBU, 4%DBU electrolytes and the 0%DBU electrolyte before polymerization; j) FTIR‐ATR spectra of the four electrolytes and pure DOL; k) Raman spectra of LiFSI, LiDFOB, pure DBU, pure DOL, the 0%DBU and 4%DBU electrolytes l) and local enlarged plot; m) Schematic illustrations of the solvation structures in the commercial, 0%DBU, and 4%DBU electrolytes, as well as the bulk and interfacial properties of LFP||graphite cells in the corresponding electrolyte systems.

Molecular dynamics (MD) simulations were performed on the three electrolytes, yielding solvation structure snapshots (**Figure** **4**a–c; Figure S23, Supporting Information), along with corresponding radial distribution functions (RDF) and calculated coordination numbers (CNs) of Li^+^ (Figure 4d–f; Figure S24, Supporting Information). The first solvation shell radius for Li^+^ was selected to be 3 Å. At 25 °C, the total CN for the commercial electrolyte was 5.71, with 3.75 for the O atom (from the solvent molecule), significantly higher than 1.96 for the F atom (from PF_6_^−^). The total CN for the 0%DBU electrolyte was 5.36, with the CN for the O atom from PDOL being 2.42, accounting for the largest proportion. For the 4%DBU electrolyte, the total CN was 5.28, with the O atom (from DOL) CN of 2.36, and the rest from the anions. These results indicate that inhibiting DOL polymerization significantly promoted the participation of anions such as FSI^−^ and DFOB^−^ in the Li^+^ solvation structure. Highly similar results were obtained under high and low‐temperature conditions of ±60 °C. Furthermore, the Li^+^ first solvation shell solvation structures of the three electrolytes at different temperatures were statistically analyzed and classified into solvent‐separated ion pairs (SSIP, anion/Li^+^ coordination number = 0), contact ion pairs (CIP, anion/Li^+^ coordination number = 1), and aggregates (AGG, anion/Li^+^ coordination number > 1).^[^ ^42^ ^]^ Figure 4g compares the proportions of SSIP, CIP, and AGG for the three electrolytes at −60, 25, and 60 °C. The 4%DBU electrolyte had an AGG proportion of ≈60%, significantly higher than that of 0%DBU (≈40%) and commercial electrolytes (≈25%). Numerous studies have reported that anion‐rich AGG solvation structures aid in weakening the coordination between Li^+^ and solvent molecules and accelerating the desolvation process,^[^ ^43^ ^]^ thereby significantly promoting the diffusion and migration of Li^+^ in the electrolyte bulk phase and manifesting as an increase in the ionic conductivity and Li^+^ transference number (Figure 1d,e). Moreover, since the formation of SEI and CEI primarily depends on the first solvation shell of Li^+^, the rich‐AGG structure not only reduces the desolvation energy barrier at the interfaces but also effectively suppresses solvent decomposition, promoting the formation of SEI and CEI rich in inorganic substances (Figures 2h and 3h).^[^ ^43^, ^44^ ^]^ This concurrently enhances interface stability and significantly accelerates the Li^+^ transport process across EEIs, manifesting macroscopically reduced charge transfer and Li^+^ transport activation energies in SEI/CEI (Figures 2a,b and 3a,b). Figure 4h and Figure S25 (Supporting Information) illustrate the representative solvation structures of the three electrolytes, with the 4%DBU electrolyte solvation structure exhibiting generally lower desolvation energies (E_d_). For example, the E_d_ of Li^+^[DOL]_2_[FSI^−^]_2_[DFOB^−^]_1_ was −0.240 Hartree, significantly lower than that of the representative solvation structure of the commercial electrolyte Li^+^[EC]_2_[DMC]_1_[EMC]_1_[PF_6_^−^]_1_ at −0.320 Hartree. In the 0%DBU electrolyte, a significant amount of PDOL participated in the Li^+^ solvation structure, making the desolvation process more difficult. For instance, the E_d_ for the typical Li^+^[FSI^−^]_1_[PDOL] was as high as −0.618 Hartree.

To experimentally validate the solvation structures of the three electrolytes, ^7^Li NMR measurements were conducted at room temperature, which highly correlated with the computational results. As shown in Figure 4i, the 4%DBU electrolyte exhibited a significant shift towards a higher field (corresponding to a lower frequency) compared to the commercial electrolyte, indicating a denser electron cloud density around the ^7^Li nucleus and an enhanced deshielding effect,^[^ ^45^ ^]^ thereby confirming a larger proportion of anions participating in the Li^+^ solvation structure compared to the commercial electrolyte. The 0%DBU electrolyte displayed extremely low chemical shifts and a diffuse peak shape, attributed to the strong and complex interactions between Li^+^ and the PDOL. Furthermore, the ^7^Li NMR chemical shift for the 0%DBU electrolyte prior to polymerization was negligible in comparison to the 4%DBU electrolyte. Moreover, their Fourier transform infrared‐attenuated total reflectance (FTIR‐ATR) spectra exhibited substantial overlap (Figure 4j). These findings imply that the primary role of the 4 vol% DBU additive was to inhibit polymerization, with minimal impact on the electrolyte's solvation structure. The FTIR‐ATR spectrum of the 0%DBU electrolyte exhibited a marked attenuation of the C─H out of plane at 916.2 cm^−1^, along with the appearance of long chain vibration peaks at 748.4 and 835.3 cm^−1^, suggesting substantial DOL polymerization.^[^ ^25^ ^]^ The Raman spectrum of the 0%DBU electrolyte, shown in Figure 4k, exhibited peaks ≈840 cm^−1^, corresponding to CH_2_ rocking and C─O vibrations, which were attributed to the long chain of PDOL. The enlarged Raman spectrum detail in Figure 4k revealed a blue shift for the C─O─C bending peak of DOL upon the introduction of LiFSI and LiDFOB. Notably, the 4%DBU electrolyte exhibited a more pronounced blue shift than the 0%DBU electrolyte, suggesting an increased dominance of CIP and AGG in its solvation structure. Figure S26a (Supporting Information) demonstrates a shift of the C─O stretching peak to higher energy, further substantiating the more pronounced interactions between DOL and LiFSI/LiDFOB within the 4%DBU electrolyte.^[^ ^46^ ^]^

Integrating the experimental and computational analyses, the 4%DBU electrolyte, characterized by its unique weak solvation structure with a high content of anion‐rich AGG, exhibited low desolvation energies and promoted the formation of inorganic‐rich SEI and CEI. Macroscopically, this enhanced the kinetic process of Li^+^ transport across the electrolyte bulk and EEIs within a wide temperature range, while also improving the electrochemical stability of the interface. These characteristics are crucial for enhancing the wide‐temperature and high‐rate performance of LIBs. (Figure 4m)

### 2.4 Electrochemical Performance of Graphite||Li and LFP||Li Half Cells

**Figure 5:**
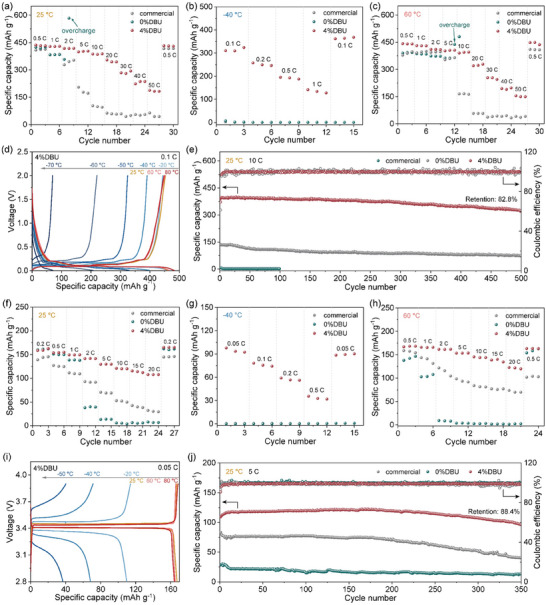
Electrochemical performance of graphite||Li and LFP||Li half cells. Rate capabilities at a) 25 °C, b) −40 °C, and c) 60 °C for graphite||Li half cells with the commercial, 0%DBU, and 4%DBU electrolytes; d) specific capacity‐voltage profiles for the graphite||Li half cell with the 4%DBU electrolyte at different temperatures; e) cycling performance of graphite||Li half cells with the three electrolytes at 25 °C at 10 C. Rate capabilities at f) 25 °C, g) −40 °C, and h) 60 °C for LFP||Li half cells with the three electrolytes; i) specific capacity‐voltage profiles for the LFP||Li half cell with the 4%DBU electrolyte at different temperatures; j) cycling performance of LFP||Li half cells with the three electrolytes at 25 °C at 5 C.

To validate the effectiveness of the DBU‐inhibited DOL polymerization strategy in enhancing the electrochemical performance of LIBs, half cells comprising graphite and LFP electrodes were assembled and tested for charge‐discharge performance across a wide temperature range. Additionally, Figure S29 (Supporting Information) depicts the electrochemical windows of three electrolytes, highlighting that the 4%DBU electrolyte maintained an oxidation potential over 3.85 V across 25, −40, and 60 °C, ideal for LFP cathode compatibility. **Figure** **5**a–c illustrates the rate capabilities of graphite||Li half cells with the commercial, 0%DBU, and 4%DBU electrolytes at 25, −40, and 60 °C (Figures S30a–g, Supporting Information display the specific capacity‐voltage curves). Notably, at lower currents, the graphite||Li half cells exhibited a phenomenon exceeding the theoretical specific capacity (372 mAh g^−1^), attributed to the use of nano‐sized graphite (with a platelet diameter of ≈400 nm and a thickness of < 40 nm) instead of the more common micron‐sized graphite. The additional specific capacity was contributed by the additional lithium storage sites provided by the large specific surface area (Figure S28, Supporting Information). The graphite||Li half cell with the 4%DBU electrolyte demonstrated superior rate capabilities across the wide temperature range, maintaining high reversible specific capacities of 187.9 mAh g^−1^ at 25 °C and 155.0 mAh g^−1^ at 60 °C with a high rate of 50 C (1 C = 372 mA g^−1^). In contrast, the graphite||Li half cell with the commercial electrolyte showed a rapid decline in specific capacity when the rate was increased to 5 C at 25 °C and 10 C at 60 °C. The graphite||Li half cells with the 0%DBU electrolyte exhibited abnormal overcharge at moderately increased rates (25 °C at 2 C and 60 °C at 5 C), which was likely due to the slow Li^+^ transport kinetics of the polymer chains leading to the deterioration of the voltage hysteresis effect at high currents.^[^ ^47^ ^]^ At low temperatures of −40 °C, the 4%DBU electrolyte system exhibited a more significant advantage. Specifically, the cell with the 4%DBU electrolyte exhibited a high reversible specific capacity of 310.5 mAh g^−1^ at 0.1 C and maintained 141.7 mAh g^−1^ at 1 C, while the cells in the commercial and 0%DBU electrolyte systems showed negligible specific capacity due to the difficulty in Li^+^ transport at −40 °C. Figure 5d presents the specific capacity‐voltage curves of the graphite||Li half cell with the 4%DBU electrolyte at −70, −60, −50, −40, −20, 25, 60, and 80 °C using a 0.1 C rate, with corresponding reversible specific capacities reaching 69.5, 220.2, 324.4, 390.8, 444.0, 452.6, 451.3, and 447.9 mAh g^−1^, significantly superior to those of the commercial and 0%DBU electrolyte systems (Figures S30h,i, Supporting Information). To further assess the stability of different electrolyte systems with graphite anodes, the cycling performance at various temperatures was tested. The graphite||Li half cell containing the 4%DBU electrolyte exhibited an ultra‐high specific capacity retention rate of 96.3% after 900 cycles of 0.5 C at room temperature (Figure S30j, Supporting Information). At a high rate of 10 C, the graphite||Li half cell with the 4%DBU electrolyte system maintained a reversible specific capacity of 323.4 mAh g^−1^ after 500 cycles, with a capacity retention of 82.8%. In contrast, the graphite||Li half cell with the commercial electrolyte system retains only 72.4 mAh g^−1^ of reversible specific capacity, while the graphite||Li half cell with the 0%DBU electrolyte system exhibited a capacity close to zero at 10 C rate (Figure 5e). At a low temperature of −40 °C and a high temperature of 60 °C, the graphite||Li half cells with 4%DBU electrolyte also demonstrated superior reversible specific capacity and excellent cycling stability (Figures S30k,l, Supporting Information).

Similarly, the wide‐temperature electrochemical performance of LFP half cells was tested with the three electrolytes. At 25 and 60 °C, the LFP half cells with the commercial electrolyte retained reversible specific capacities of 29.3 and 69.9 mAh g^−1^ at 20 C (1 C = 170 mA g^−1^); and the cells with the 0%DBU electrolyte exhibited a rapid decay in specific capacity when the rate exceeds 2 C. At −40 °C, the LFP||Li half cells with both the commercial and 0%DBU electrolytes exhibited near‐zero specific capacities. In contrast, the LFP||Li half cell with the 4%DBU electrolyte maintained ultra‐high reversible specific capacities of 107.7 (20 C), 35.6 (0.5 C), and 122.8 (20 C) mAh g^−1^ at 25, −40, and 60 °C (Figure 5f–h). At 0.05 C, the reversible specific capacities of the LFP||Li half cells were further tested at −50, −40, −20, 25, 60, and 80 °C. The 4%DBU electrolyte for LFP||Li half cell exhibited reversible specific capacities of 36.8, 68.3, 109.8, 167.2, 165.2, and 164.1 mAh g^−1^, respectively, significantly superior to those of the commercial and 0%DBU electrolyte systems (Figures S31h,i, Supporting Information). To further test the stability of the three electrolytes for the LFP cathode system, the LFP||Li half cells were cycled at −40, 25, and 60 °C (Figure 5j; Figure S31j,k, Supporting Information). With the 4%DBU electrolyte, the cell exhibited significantly superior cycling performance compared to the commercial and 0%DBU electrolytes at all temperatures. Particularly at room temperature with 5 C for 350 cycles, the LFP||Li half cell with the 4%DBU electrolyte system displayed an ultra‐high reversible specific capacity of nearly 100 mAh g^−1^ with an 88.4% retention, whereas the commercial and 0%DBU electrolyte systems retained only 40.6 and 13.1 mAh g^−1^, respectively. The exceptional stability of the battery with the 4%DBU electrolyte was closely associated with the stable and efficient Li^+^‐conducting inorganic‐rich CEI formed on the LFP surface.

### 2.5 Electrochemical Performance of LFP||Graphite and LFP||Li Full Cells

**Figure 6:**
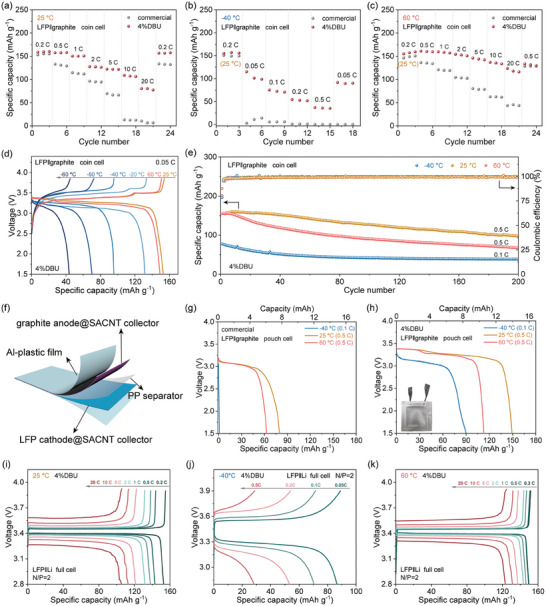
Electrochemical performance of LFP||graphite and LFP||Li full cells. Rate capabilities of LFP||graphite coin cells at a) 25 °C, b) −40 °C, and c) 60 °C with the commercial and 4%DBU electrolytes; d) specific capacity‐voltage profiles for the LFP||graphite coin cell with the 4%DBU electrolyte at different temperatures; e) cycling performance of the LFP||graphite coin cell with the 4%DBU electrolyte at −40 °C, 25 °C, and 60 °C. f) Schematic illustration of the LFP||graphite pouch cell structure; specific capacity‐voltage curves for the LFP||graphite pouch cell with the g) commercial electrolyte and h) 4%DBU electrolyte at −40 °C, 25 °C, and 60 °C. Rate capability curves of LFP||Li full cells at i) 25 °C, j) −40 °C, and k) 60 °C with the 4%DBU electrolytes.

Full cells comprising LFP and graphite electrodes were assembled to further validate the practical effectiveness of the DOL‐based electrolyte system with DBU additive in enhancing the wide‐temperature performance of LIBs. **Figure** **6**a–c respectively depict the rate capabilities of LFP||graphite coin cells with the commercial and 4%DBU electrolytes at 25, −40, and 60 °C. At 25 °C, the cell with the 4%DBU electrolyte maintained a high reversible specific capacity of 80.3 mAh g^−1^ at 20 C, which was 50.6% of that at 0.2 C, whereas the commercial electrolyte system only achieved 6.6 mAh g^−1^, which was 4.2% of that at 0.2 C. At the low temperature of −40 °C, the full cell with the commercial electrolyte exhibited a very low reversible capacity (close to 0) even at a small rate of 0.05 C, whereas the cell with 4%DBU electrolyte showed a high reversible specific capacity of 101.2 mAh g^−1^, retaining 36.9 mAh g^−1^ even at 0.5 C. At 60 °C, the full cell with 4%DBU electrolyte also displayed excellent rate capabilities, achieving a high reversible specific capacity of 118.0 mAh g^−1^ at 20 C, significantly higher than the 43.8 mAh g^−1^ for the commercial electrolyte. Moreover, under the various temperature conditions mentioned above, when transitioning the test rate from high to low, the reversible specific capacity of the full cell with the 4%DBU electrolyte could fully recover, demonstrating its excellent reversibility. To more thoroughly evaluate the adaptability of the designed electrolyte system across a wide temperature range, additional charge‐discharge tests were conducted at temperatures ranging from −60 °C to 60 °C at 0.1 C (Figure 6d). At −60, −50, −40, −20, 25, and 60 °C, the full cell with the 4%DBU electrolyte exhibited reversible capacities of 43.6, 70.2, 95.4, 131.4, 152.9, and 149.0 mAh g^−1^. To further validate its stability across a wide temperature range, the cycling performance was tested at 25 °C, −40 °C, and 60 °C (Figure 6e). The full cell with the 4%DBU electrolyte could stably cycle for 200 cycles. Subsequently, LFP||graphite pouch cells were assembled to demonstrate the effectiveness of the electrolyte strategy at a larger scale (Figure 6f). As shown in Figure 6g,h, the pouch cells with the commercial and 4%DBU electrolytes exhibited reversible capacities of 79.8 and 149.3 mAh g^−1^ at 25 °C (0.5 C), 62.7 and 112.9 mAh g^−1^ at 60 °C (0.5 C), and 0.94 and 90.2 mAh g^−1^ at −40 °C (0.1 C), respectively. Beyond this, the 4%DBU electrolyte also exhibited high‐rate performance at wide temperatures within the SiC anode system, confirming its applicability across various LIBs (Figure S33, Supporting Information). Furthermore, LFP||Li full cells were assembled to evaluate the suitability of the 4%DBU electrolyte in lithium metal battery (LMB) systems. The cells demonstrated superior rate performance across a broad temperature range, as depicted in Figure 6i–k. At 25, −40, and 60 °C, the cells delivered reversible specific capacities of 105.7 mAh g^−1^ at 20 C, 28.8 mAh g^−1^ at 0.5 C, and 124.4 mAh g^−1^ at 20 C, respectively. Moreover, the electrolyte designed in this study offers a competitive edge over reported LIB performance in existing literature, particularly in adaptability to diverse thermal conditions and capacity retention under rapid charging–discharging (Table S3, Supporting Information). Finally, the comparison with previously reported PDOL‐based electrolyte systems for LMBs further confirms the remarkable superiority of the DBU inhibition strategy for preventing DOL‐based electrolyte polymerization, significantly enhancing the battery performance across a wide temperature range and at high rates (Table S4, Supporting Information).

## 3 Conclusion

In this work, we delve into the micro‐mechanism of the ring‐opening polymerization reaction induced by common lithium salts such as LiFSI and LiDFOB in DOL. By introducing the basic organic additive DBU, we successfully inhibit the polymerization process. This breakthrough addresses the long‐standing issue of limited compatibility between DOL and lithium salts, unleashing the advantages of DOL's weak Li^+^‐solvent binding energy at the microscopic level and its wide liquid phase temperature range at the macroscopic level. As a result, the application scenarios of DOL in LIBs across extreme temperatures are significantly broadened. Specifically, the developed electrolyte, composed of 0.8 m LiFSI and 0.2 m LiDFOB in DOL with 4 vol% DBU, forms an anion‐dominated weak solvation structure devoid of interference from the PDOL polymer chains. Therefore, this electrolyte demonstrates significant advantages across a wide temperature range in both the bulk phase and EEIs, including the ultra‐high ionic conductivity, the high Li^+^ transference number, the robust SEI/CEI rich in inorganic components, as well as the significantly reduced activation energy barriers for interfacial charge and Li^+^ transfer. Utilizing the developed electrolyte within the LFP||graphite battery system results in remarkable high‐rate performance across an extensive temperature spectrum. The specific capacities achieved are 101.2 mAh g^−1^ at room temperature (20 C), 36.9 mAh g^−1^ at −40 °C (0.5 C), and 118.0 mAh g^−1^ at 60 °C (20 C). These findings offer pivotal insights for the advancement of wide‐temperature rapid‐charging electrolytes.

## Conflict of Interest

The authors declare no conflict of interest.

## Supporting information



Supporting Information

## Data Availability

The data that support the findings of this study are available from the corresponding author upon reasonable request.
