# Supplementary material for: 1,8‐Diazabicyclo[5.4.0]undec‐7‐ene as Cyclic Ether Electrolyte Polymerization Inhibition for Wide‐Temperature‐Range High‐Rate Lithium‐ion Batteries
Source: Adv Sci (Weinh). 2024 Nov 8;12(2):2409259. doi: 10.1002/advs.202409259 (PMC11727236; doi:10.1002/advs.202409259)
Supplement: Supplementary file 1 — Supporting Information [file ADVS-12-2409259-s001.docx]

Supporting Information

1,8-Diazabicyclo[5.4.0]undec-7-ene as cyclic ether electrolyte polymerization inhibition for wide-temperature-range high-rate lithium-ion batteries

Hui Tian, Zixin Hong, Zhenhan Fang,* Yufeng Luo,* Hengcai Wu,^[a]^ Fei Zhao, Qunqing Li, Shoushan Fan, and Jiaping Wang*

**Experimental Section**

**Preparation of electrolyte:** The commercial electrolyte (1 M LiPF_6_ in ethylene carbonate (EC): dimethyl carbonate (DMC): ethyl methyl carbonate (EMC) = 1:1:1 by volume) and lithium bis(fluorosulfonyl)imide (LiFSI) were purchased from Suzhou Dodo Chemical Technology Co. Lithium difluorooxalate borate (LiDFOB), 1,3-dioxolane (DOL) and 1,8-Diazabicyclo[5.4.0]undec-7-ene (DBU) purchased from Shanghai Aladdin Biochemical Technology Co., Ltd. The 0%DBU electrolyte was prepared by dissolving 0.8 M LiFSI and 0.2 M LiDFOB into DOL. The 4%DBU electrolyte was prepared by dissolving 0.8 M LiFSI and 0.2 M LiDFOB into DOL with 4 vol% DBU. All electrolytes were stored in an Argon (Ar)-filled glove box (H_2_O and O_2_ < 0.1 ppm).

**Preparation of electrodes**: The graphite and LiFePO_4_ (LFP) were purchased from Hefei Kejing Material Technology Co., Ltd. The graphite (or LFP/SiC) electrode slurry was prepared by mixing 80 wt% graphite (or LFP/SiC) powder, 10 wt% Super P, and 10 wt% polyvinylidene fluoride (PVDF) in N-methyl pyrrolidine (NMP) solvent. Then the obtained slurry was homogeneously coated on a 20-layer crossover membrane of Super-aligned Carbon Nanotubes ^[1]^ (SACNTs) as current collectors and transferred into a vacuum oven for drying overnight. Compared to metal current collectors, the SACNTs current collectors exhibited superior wettability, stronger adhesion, and lower contact resistance at the interface with electrodes, thereby enabling efficient electron transfer and enhancing rate performance. Figure S1 illustrates SEM images of the interfaces between electrode plates and both metallic and SACNTs-based current collectors, highlighting a notably closer contact with SACNTs. These phenomena have been rigorously examined and characterized in our prior research endeavors. ^[2]^ The final electrodes were punched into 10 mm disks (for coin cells) and 4 cm *5 cm squares (for pouch cells) for both the anodes and the cathodes. The areal mass loadings for the graphite anode, SiC anode, and LFP cathode were 2.2-2.5, 2.5-2.8, and 5.5-6.3 mg cm^-2^, respectively.

**Electrochemical measurements:** The CR2025 coin cells were assembled in an Ar-filled glove box for the electrochemical measurements. The graphite (or LFP/SiC)||Li half coin cells were assembled by lithium foil, Celgard separator and graphite (or LFP/SiC) electrode with 70 μL electrolytes. The current densities corresponding to 1 C were 372 mA g^-1^ for the graphite electrode, 650 mA g^-1^ for the SiC electrode and 170 mA g^-1^ for the LFP electrode in the half cells, respectively. The test voltage ranges were 0.01–2 V for graphite||Li and SiC half cells and 2.8–3.9 V for LFP||Li half coin cells. The LFP||graphite full coin cells and full pouch cells were assembled by graphite anodes, Celgard separators and LFP cathodes with electrolytes. The negative/positive capacity ratio was about 1.1:1, the charge and discharge voltage range were 1.5-3.85 V for coin cells and 1.5-3.75 V for pouch cells, and the C-rate was calculated based on the mass of the LFP cathode (1 C=170 mA g^-1^). The graphite anodes were subjected to an electrochemical pre-lithiation treatment before the assembly of the full cells. This involved initially assembling the graphite half-cells, which were then cycled for five rounds at 0.5 C before disassembly to obtain the pre-lithiated anodes. Notably, due to the gelation of the 0%DBU electrolyte, it was challenging to completely separate the separators from the electrodes while maintaining the integrity of the electrodes during the disassembly of the pre-lithiated half-cells. Consequently, the full cell systems were assembled and tested for comparison only with the commercial and 4%DBU electrolyte systems. The lithium anode in the LFP||Li full cell was obtained by electrochemically depositing lithium onto Cu foil to a quantified amount, resulting in a full cell with an N/P ratio of 2. All the cells were maintained on an open circuit for 2 hours before the electrochemical characterization. All the galvanostatic charge/discharge measurements were examined using the LAND battery tester system (CT2001A, Wuhan, China) in the high and low temperature test chamber with controlled temperature except for the room temperature test.

Electrochemical impedance spectroscopy (EIS) of cells was measured by an electrochemical workstation at a frequency range from 0.01 to 10^6^ Hz using an amplitude of 10 mV. The data of EIS were fitted accordingly by using Zview software.

The ionic conductivity (𝜎) was tested in stainless steel (SS)||SS cells based on the following equation:

$$\begin{aligned} \sigma=\frac{L}{R_{b}A}\#\left( \text{Equation 1} \right) \end{aligned}$$

where *L*, *R*_b_ and *A* were the distance between two electrodes, the electrolyte resistance and the electrode area, respectively.

The Li^+^ transference number ($t_{{Li}^{+}}$) was tested in Li||Li cells by combing EIS measurements and constant voltage polarization (10 mV) based on the following equation:

$$\begin{aligned} t_{{Li}^{+}}=\frac{I_{ss}\left( \Delta V-I_{0}R_{0} \right)}{I_{0}\left( \Delta V-I_{ss}R_{ss} \right)}\#\left( \text{Equation 2} \right) \end{aligned}$$

where $I_{0}$, $I_{ss}$, $R_{0}$, $R_{ss}$, $\Delta V$ were the initial current, the steady state current, the initial interfacial resistance, the steady state interfacial resistance and the polarization voltage applied, respectively.

The activation energy ($E_{a}$) of the charge transfer and Li^+^ transport in SEI (or CEI) processes was obtained according to the following equation:

$$\begin{aligned} \frac{1}{R_{\left( ct, SEI \right)}}=Ae^{-\frac{E_{a}}{RT}}\#\left( \text{Equation 3} \right) \end{aligned}$$

where $R_{(ct, SEI)}$ was the charge transfer resistance or the Li^+^ transport resistance in SEI, $A$is the pre-exponential constant, $R$is the gas constant, $T$ is the absolute temperature.

Linear sweep voltammetry (LSV) was performed on SS||Li cells, scanning from the open-circuit potential to 0 or 6 V vs. Li^+^/Li at a scan rate of 1 mV s^-1^.

**Characterization:** The nuclear magnetic resonance (NMR) spectra were obtained using a JNM-ECA600 model 600 MHz NMR spectrometer. For the acquisition of the ^1^H NMR spectrum, deuterated dimethyl sulfoxide (DMSO-d6) was used as the deuterated solvent. In the case of the ^7^Li NMR spectrum, a 1 M lithium chloride (LiCl) solution in deuterated water (D_2_O) was employed for external field calibration. The viscosities of the electrolytes were measured with an Anton Paar ViscoQC 300-L viscometer. The differential scanning calorimetry (DSC) measurements were conducted on a TA DSC250 instrument, heating from -120.0 °C to 100.0 °C at 10.0 °C min^-1^ under a nitrogen atmosphere. The Raman spectra were measured by a HORIBA LabRAM HR laser Raman spectrometer. Raman CW (continuous wave) measurements with laser wavelength of 633 nm were run with a HORIBA LabRAM HR laser Raman spectrometer with a microscope focusing on the sample via 50× objective. The Fourier transform infrared-attenuated total reflectance (FTIR-ATR) measurements were conducted using the Nicolet iS50 model instrument from Thermo Fisher Scientific.

X-ray photoelectron spectroscopy (XPS) data were acquired using the state-of-the-art Quantera II instrument from ULVAC-PHI with Al Kα radiation. Graphite electrodes for XPS were sourced from half-cells cycled at 0.5 C for five cycles in different electrolytes at room temperature, while LFP electrodes were retrieved from similar cycling at 0.2 C in various electrolytes. Except for the partial electrodes retaining surface polymer in the 0%DBU electrolyte system, the remaining samples were surface-cleaned with dimethyl ether (DME) and dried overnight within the Ar-filled glove box before testing. The binding energy was corrected based on the C1s spectrum, assigning C-C to 284.8 eV.

Microscopic morphology was characterized using a scanning electron microscope (SEM, Sirion 200, FEI). The graphite anodes were harvested from graphite half cells exposed to various electrolyte systems, following cycling at room temperature at rates of 0.5, 1, 2, 5, 10, 20, 30, 40, 50, and 0.5 C for three cycles each. Additionally, cycling was conducted at -40 °C at rates of 0.1, 0.2, 0.5, 1, and 0.1 C for three cycles each, and at 60 °C at rates of 0.5, 1, 2, 5, 10, 20, 30, 40, 50, and 0.5 C for three cycles each, after which the batteries were disassembled and the anodes were recovered. The LFP cathodes were obtained from LFP half cells subjected to different electrolyte systems, after cycling at room temperature at rates of 0.2, 0.5, 1, 2, 5, 10, 20, and 0.2 C for three cycles each. Additionally, cycling tests were carried out at -40 °C at rates of 0.05, 0.1, 0.2, 0.5, and 0.05 C for three cycles each, and at 60 °C at rates of 0.5, 1, 2, 5, 10, 20, and 0.5 C for three cycles each. The cathodes were then retrieved from the disassembled batteries following these cycling protocols.

**Calculations**

**Molecular dynamics simulations:** Molecular dynamics (MD) simulations were conducted utilizing the Forcite module within Materials Studio 2023 to investigate various electrolyte systems across a range of temperatures. The commercial electrolyte formulation comprised 125 Li^+^, 125 PF_6_^-^, 625 EC, 490 DMC and 404 EMC molecules. The electrolyte system labeled as “0%DBU” consisted of 125 Li^+^, 100 FSI^-^, 25 DFOB^-^, 549 DOL molecules, and 62 PDOL chains, each chain being a polymer of 20 DOL monomers. The “4%DBU” electrolyte model included 125 Li^+^, 100 FSI^-^, 25 DFOB^-^, and 1789 DOL molecules. For all MD simulations, the COMPASS III force field ^[3]^ was employed, with a time step of 1.0 femtosecond (fs). To assess the integrity of the force field parameters, an initial 50 picoseconds (ps) NPT calculation was conducted at 298.15 K, utilizing the Bussi thermostat and Berendsen barostat to maintain a pressure of 1e^-4^ GPa. Subsequent to equilibration, production runs were executed in the NVT ensemble for a duration of 20 ps. Throughout these steps, a Nosé thermostat was utilized to target temperatures of 398.15 K, 333.15 K, 298.15 K, and 213.15 K. Subsequently, 20 ps NPT simulations were conducted at 333.15 K, 298.15 K, and 213.15 K, followed by 100 ps NVT simulations at the same temperatures based on the outcomes of the previous NPT simulations. The duration of the simulations was sufficient to ensure that the electrolyte systems had reached equilibrium.

**Quantum chemistry calculations:** The de-solvation energies ($E_{d}$) of representative solvation structures for each electrolyte system, obtained from MD simulations, were calculated using the following formula:

$$\begin{aligned} E_{d}=E_{Solvation}-E_{{Li}^{+}}-x_{i}E_{{Anion}_{i}}-y_{j}E_{{Solvent}_{j}}\#\left( \text{Equation 4} \right) \end{aligned}$$

where $E_{Solvation}$, $E_{{Li}^{+}}$, $E_{{Anion}_{i}}$, $x_{i}$, $E_{{Solvent}_{j}}$, $y_{j}$ were the energy of representative solvation structure, the energy of Li^+^, the energy of each anion, the number of corresponding anions present in the solvation structure, the energy of each solvent molecule, and the number of corresponding solvent molecules within the solvation structure, respectively. All energies were computed using the DMol3 module of Materials Studio 2023, with the structures optimized utilizing the Local Density Approximation (LDA) with Perdew-Wang-91 correlation (PWC) energy functional, followed by energy calculations using the generalized gradient approximation (GGA) with Perdew-Burke-Ernzerhof (PBE) exchange-correlation functional.

Geometry optimization, frequency calculations, energy evaluations, and electronic structure analyses of electrolyte and solvent molecules were conducted using the Gaussian 16 package, employing the B3LYP functional^[4]^ and the 6-311++G(d,p) double-zeta basis set^[5]^.

The molecular orbitals, including the highest occupied molecular orbital (HOMO) and the lowest unoccupied molecular orbital (LUMO), were calculated and analyzed for LiPF_6_, LiFSI, LiDFOB, EC, DMC, EMC, and DOL (Figure S3).

The Li^+^-solvent binding energies ($E_{b})$ for EC, DMC, DEC, EMC, PC, MA, EA, MP, AN, TMP, DMSO, THF, DME and DOL were calculated (Table S1), employing the following formula:

$$\begin{aligned} E_{b}=E_{Li^{+}-solvent}-E_{{Li}^{+}}-E_{Solvent}\#\left( \text{Equation 5} \right) \end{aligned}$$

where $E_{Li^{+}-solvent}$, $E_{{Li}^{+}}$, and $E_{Solvent}$ were the single-point energies of the complex, free Li^+^, and free solvent, respectively.

The surface electrostatic potential (ESP) distribution of DBU and other organic bases was analyzed and visualized using the Multiwfn software in conjunction with Visual Molecular Dynamics (VMD) ^[6–8]^ (Figure S6 and Table S2).

The Gibbs free energy changes for the reactions of hydrated proton acid H^+^A(OH)^-^ with DOL and DBU, respectively, denoted as ΔG_1_ and ΔG_2_ (Figure 2b(ii)), were obtained using the following formula:

$$\begin{aligned} {\Delta G}_{1}=G_{DOL+H^{+}A(OH)^{-}}-G_{H^{+}A(OH)^{-}}-G_{DOL}\#\left( \text{Equation 6} \right) \end{aligned}$$

$$\begin{aligned} {\Delta G}_{2}=G_{DBU+H^{+}A(OH)^{-}}-G_{H^{+}A(OH)^{-}}-G_{DBU}\#\left( \text{Equation 7} \right) \end{aligned}$$

where $G_{DOL+H^{+}A(OH)^{-}}$, $G_{H^{+}A(OH)^{-}}$, $G_{DOL}$, $G_{DBU+H^{+}A(OH)^{-}}$, and $G_{DBU}$ were the Gibbs free energies of the combination of DOL and H^+^A(OH)^-^, H^+^A(OH)^-^, DOL, the combination of DBU and H^+^A(OH)^-^, and DBU. In particular, for ΔG_1_, “A” represented the radical produced by LiPF_6_、LiBF_4_/LiDFOB or LiFSI, while for ΔG_2_, “A” represented the radical produced by LiPF_6_、LiBF_4_/LiDFOB、LiFSI or LiTFSI.


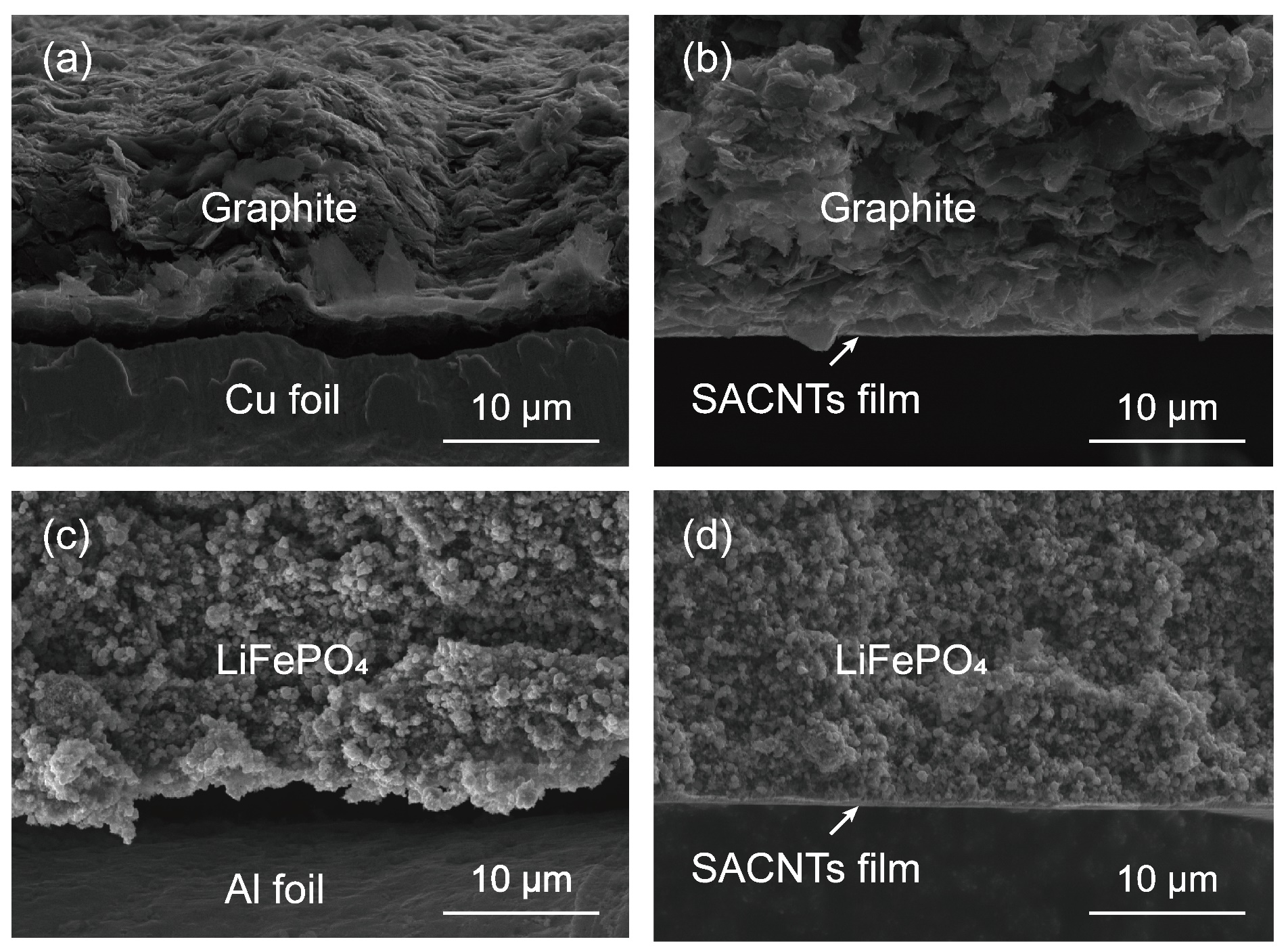


**Figure S1.** SEM images of the interfaces for the graphite anode with (a) copper (Cu) and (b) SACNTs current collectors, as well as for the LFP cathode with (c) aluminum (Al) and (d) SACNTs current collectors.

(a) LiPF_6_:


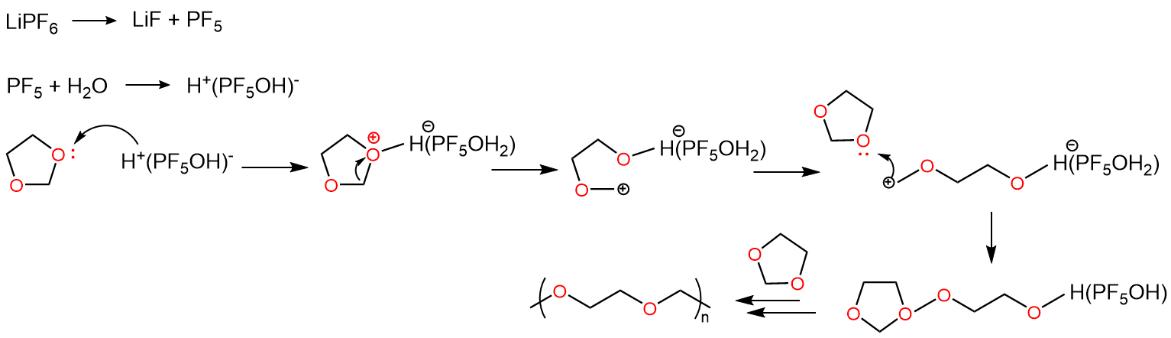


(b) LiDFOB (and LiBF_4_):


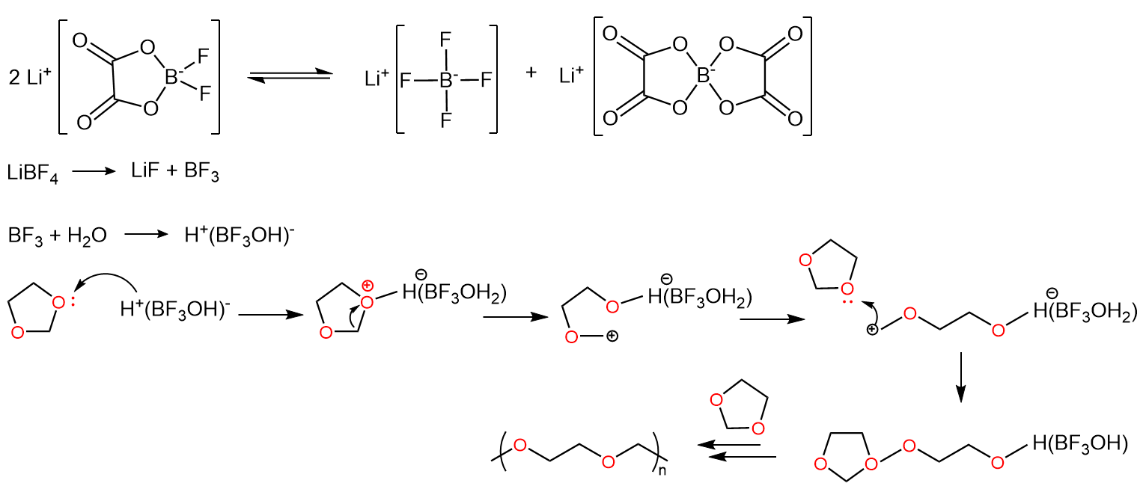


(c) LiFSI:


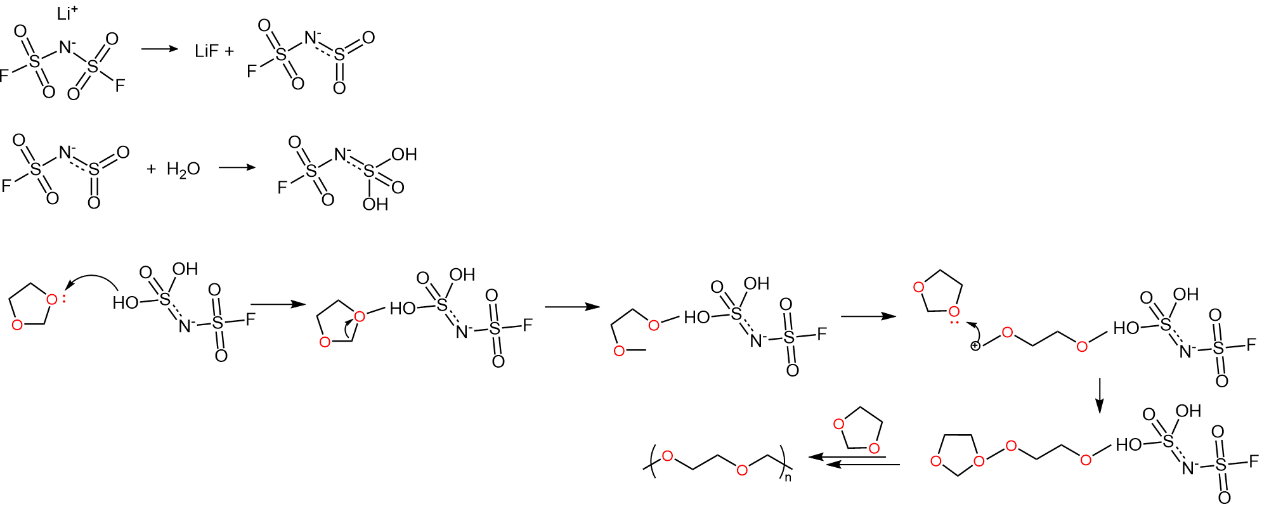


(d) “A” represents the free radicals derived from lithium salts.


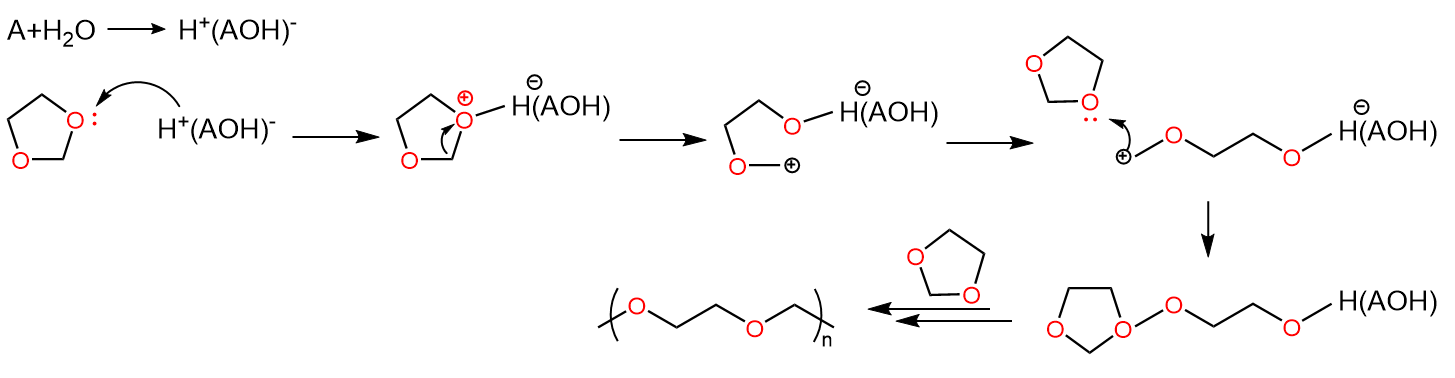


(e) LiTFSI: (No polymerization occurs)

**Figure S2.** Mechanism of DOL polymerization induced by (a) LiPF_6_; (b) LiDFOB (and LiBF_4_); (c) LiFSI; (d) General mechanism of DOL polymerization induced by lithium salts; (e) Mechanism of non-polymerization of DOL by LiTFSI.


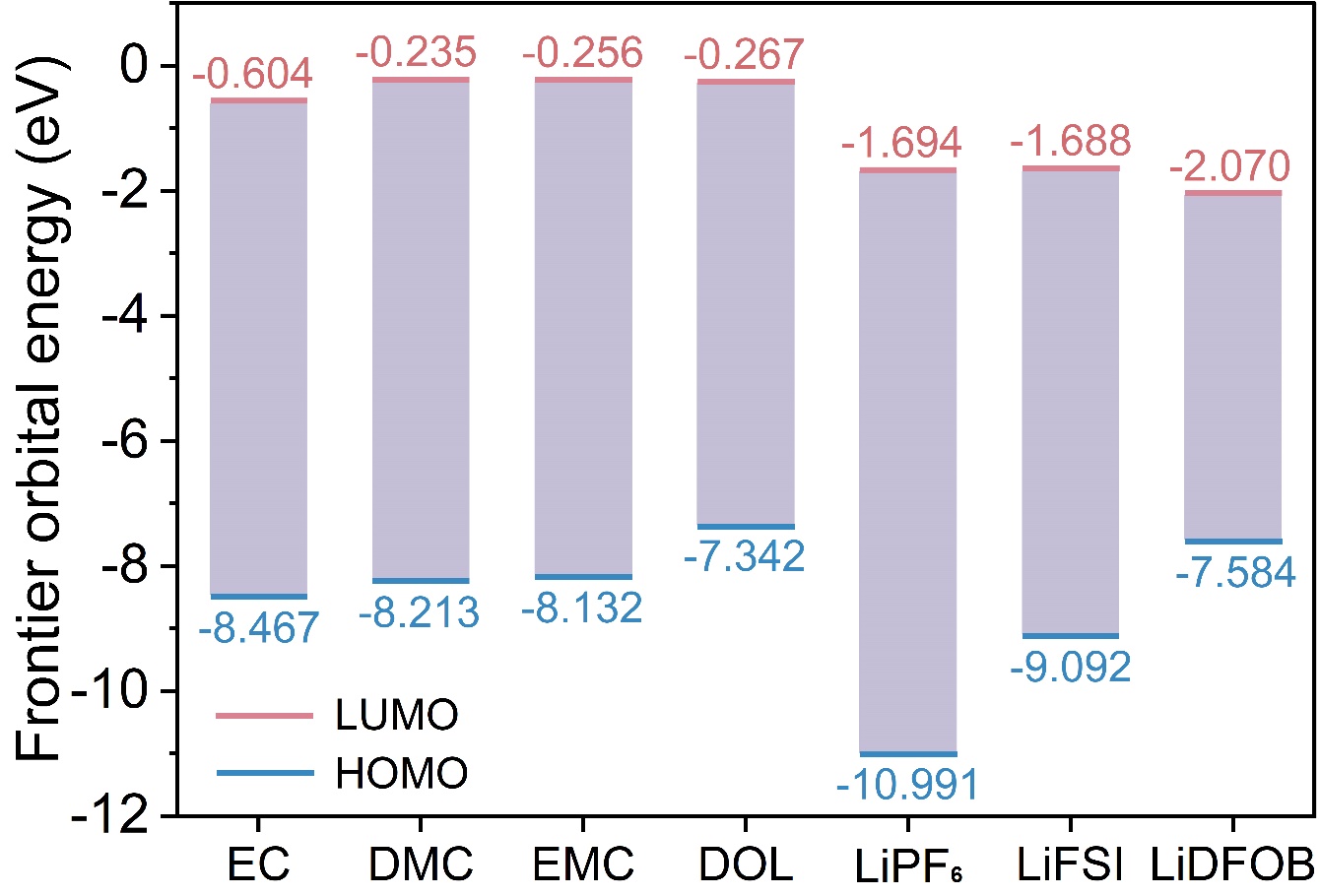


**Figure S3.** HOMO and LUMO of LiPF_6_, LiFSI, LiDFOB, EC, DMC, EMC, and DOL


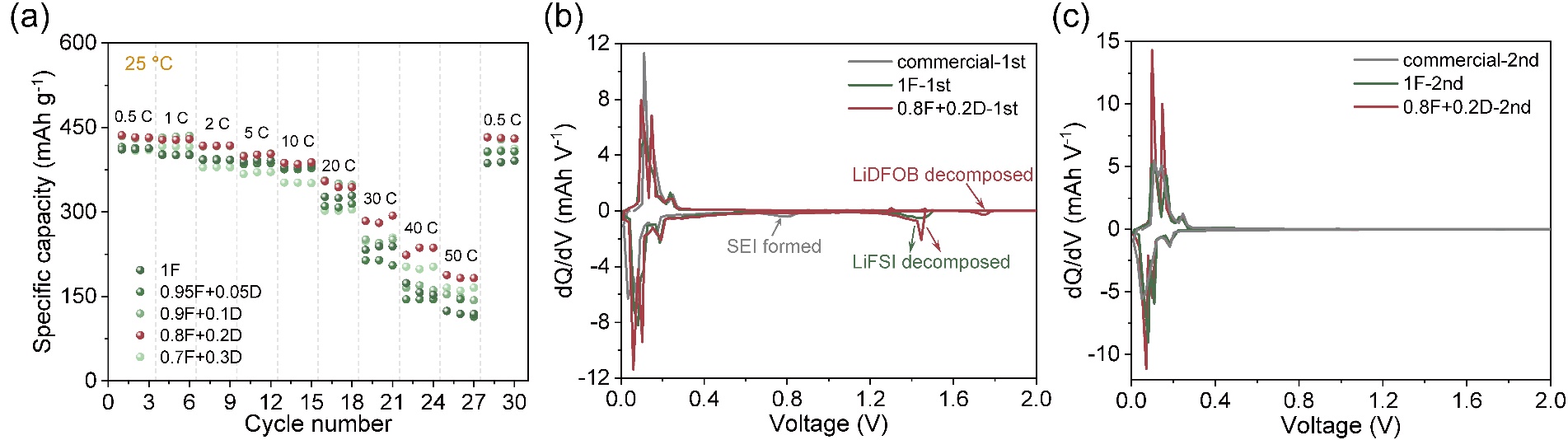


**Figure S4.** (a) Comparison of the rate capabilities of graphite half cells at room temperature under various dual-salt electrolyte systems with different salt ratios: 1 M LiFSI (denoted as “1F”), 0.95 M LiFSI + 0.05 M LiDFOB (denoted as “0.95F+0.05D”), 0.9 M LiFSI + 0.1 M LiDFOB (denoted as “0.9F+0.1D”), 0.8 M LiFSI + 0.2 M LiDFOB (denoted as “0.8F+0.2D”), and 0.7 M LiFSI + 0.3 M LiDFOB (denoted as “0.7F+0.3D”); (b) The first-cycle and (c) second-cycle dQ/dV curves for graphite half-cells in commercial, 1F, and 0.8F+0.2D electrolytes, respectively: LiDFOB decomposed preferentially, followed by LiFSI, with the film-forming properties of the 0.8F+0.2D electrolyte superior to those of the commercial electrolyte.


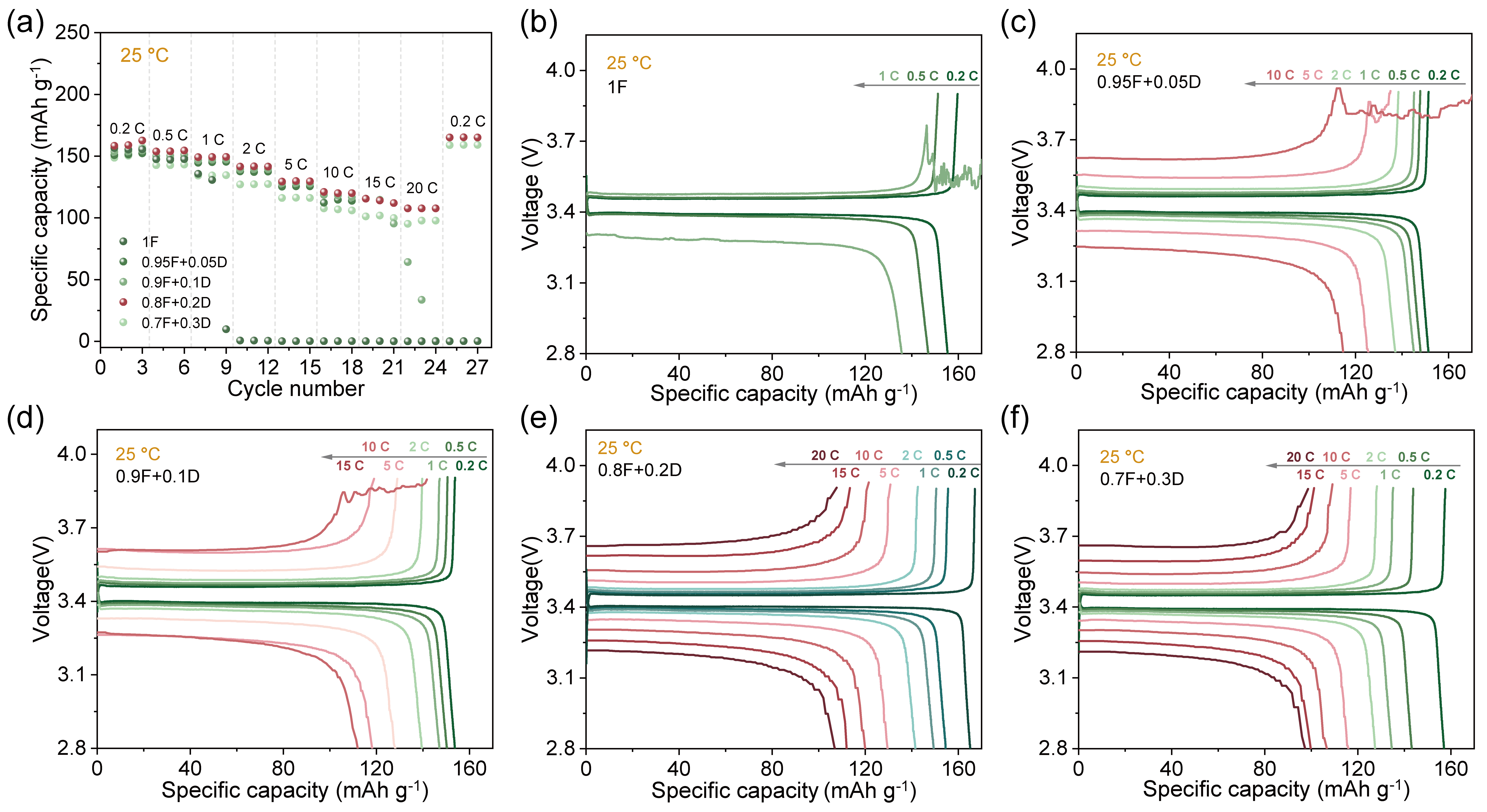


**Figure S5.** (a) Comparison of the rate capabilities of LFP half cells at room temperature under various dual-salt electrolyte systems with different salt ratios: 1F, 0.95F+0.05D, 0.9F+0.1D, 0.8F+0.2D, and 0.7F+0.3D; The specific capacity-voltage curves for LFP half-cells in the (b) 1F, (c) 0.95F+0.05D, (d) 0.9F+0.1D, (e) 0.8F+0.2D, and (f) 0.7F+0.3D electrolytes: The presence of LiDFOB is beneficial for suppressing overcharge phenomena at high rates.


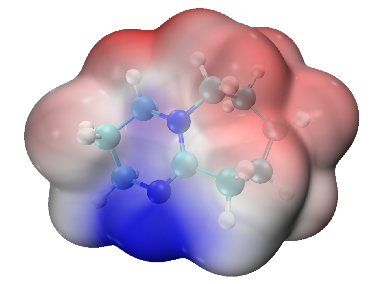


**Figure S6.** The ESP distribution of DBU, depicted with a color gradient, where red denotes areas of positive potential, indicating electron-rich zones, and blue signifies negative potential. The imino nitrogen atom in DBU shows a pronounced negative potential, suggesting it may serve as the coordination site for a hydrated proton.


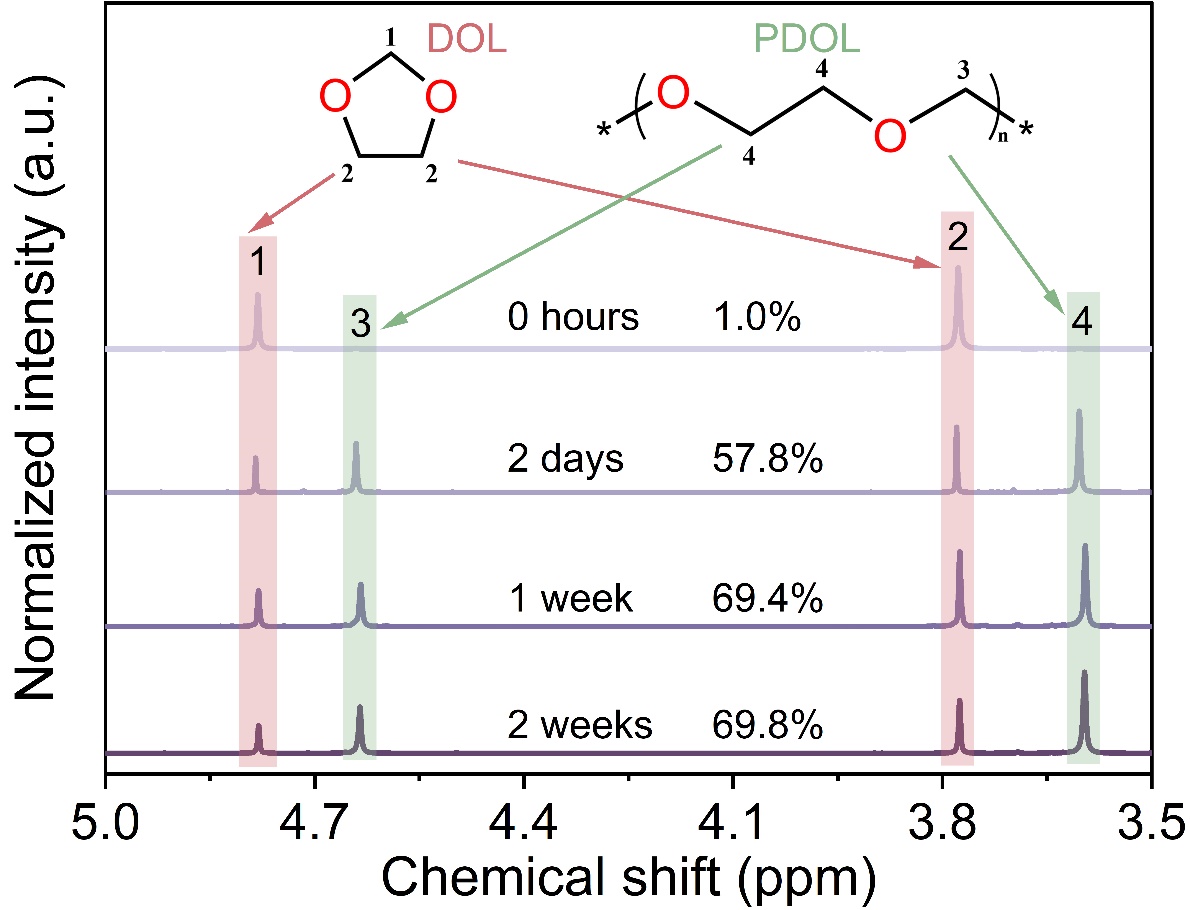


**Figure S7.** The ^1^H NMR spectra of the 0.8 M LiFSI + 0.2 M LiDFOB in DOL (DBU-free) electrolyte after different periods of standing following preparation: The polymerization process was almost saturated after 1 week of standing, which was used as the polymerization condition.


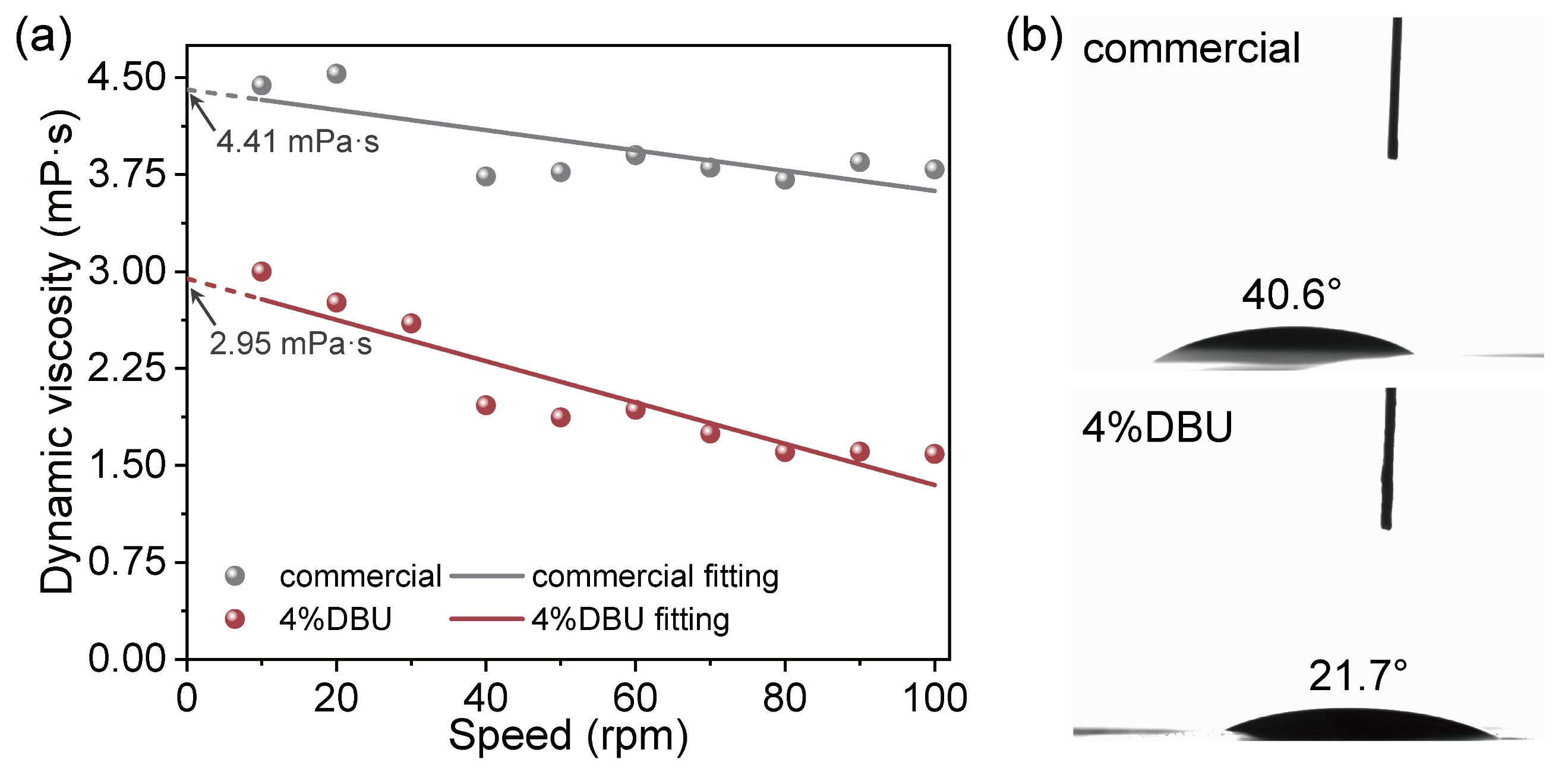


**Figure S8.** The measurements of (a) viscosity and (b) contact angle with separator for commercial and 4% DBU electrolytes at room temperature.


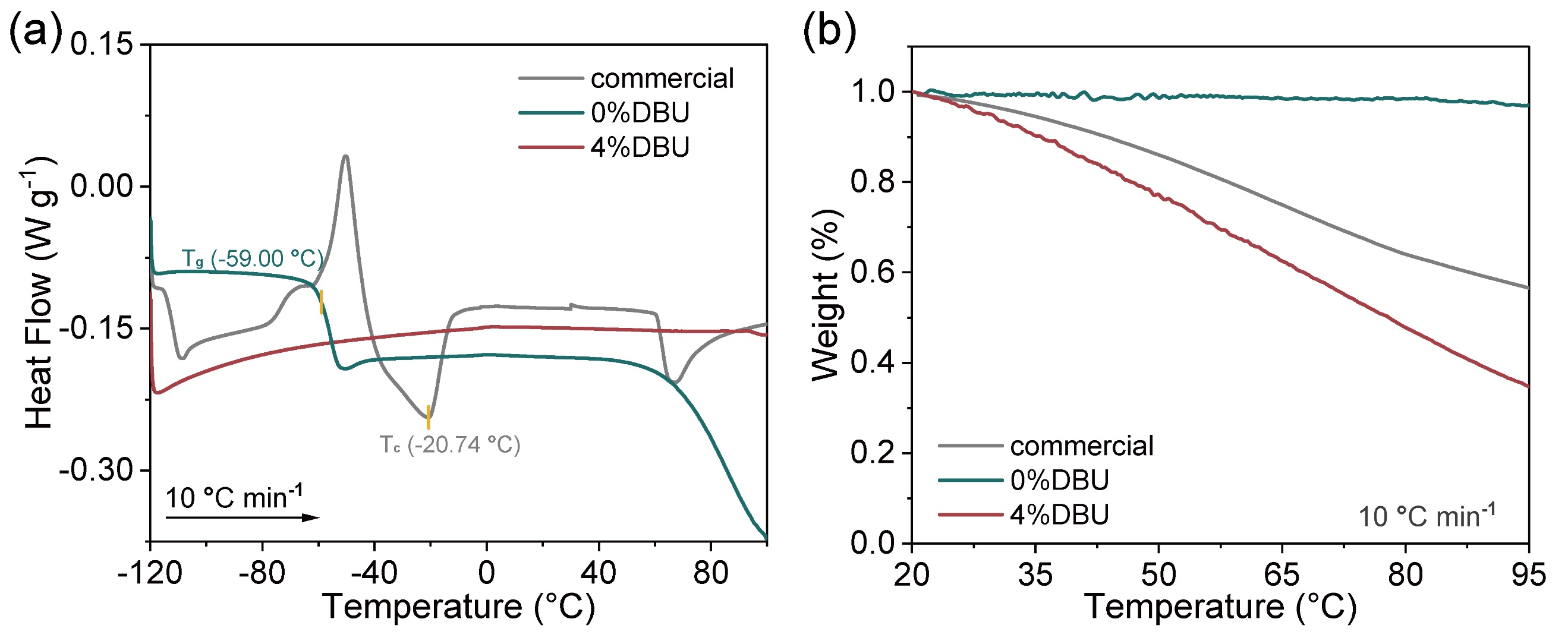


**Figure S9.** (a) DSC and (b) thermogravimetric (TG) analyses of the commercial, 0%DBU, and 4%DBU electrolytes.


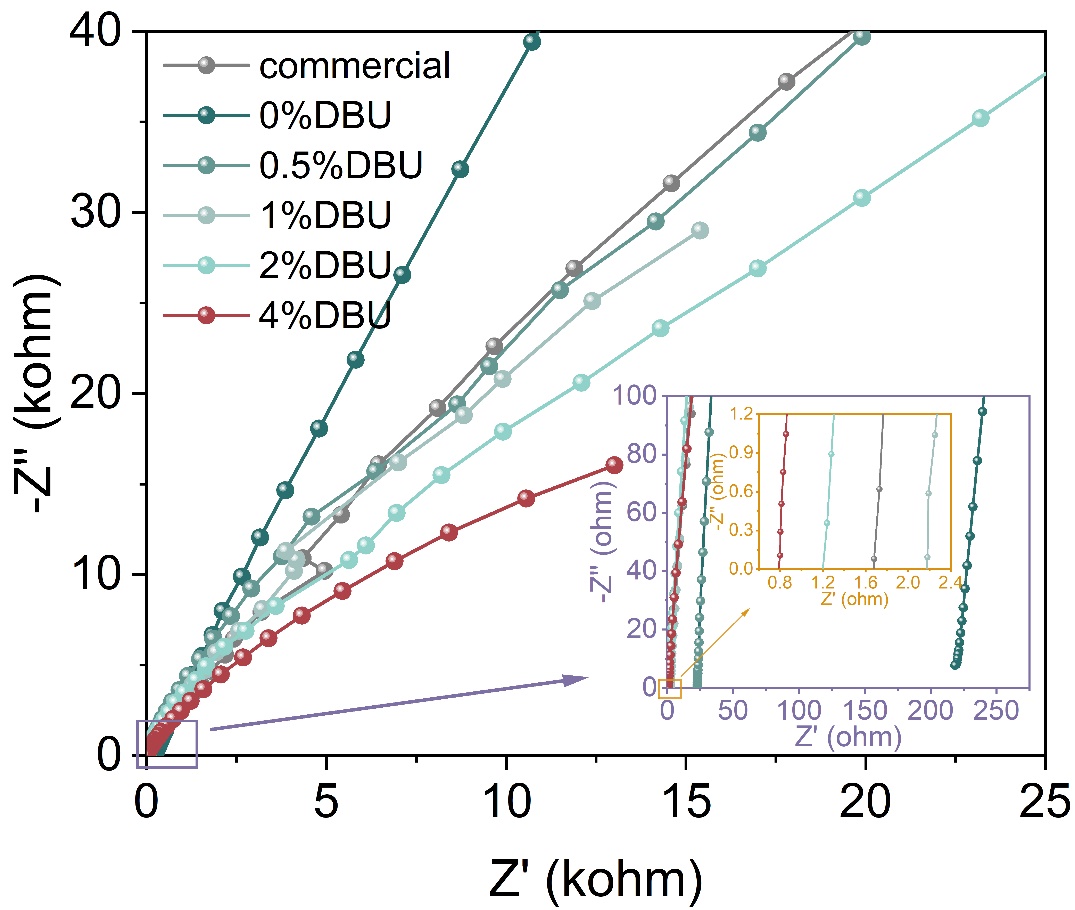


**Figure S10.** Testing of the ionic conductivity for the commercial electrolyte and DOL-based electrolytes with different DBU contents.


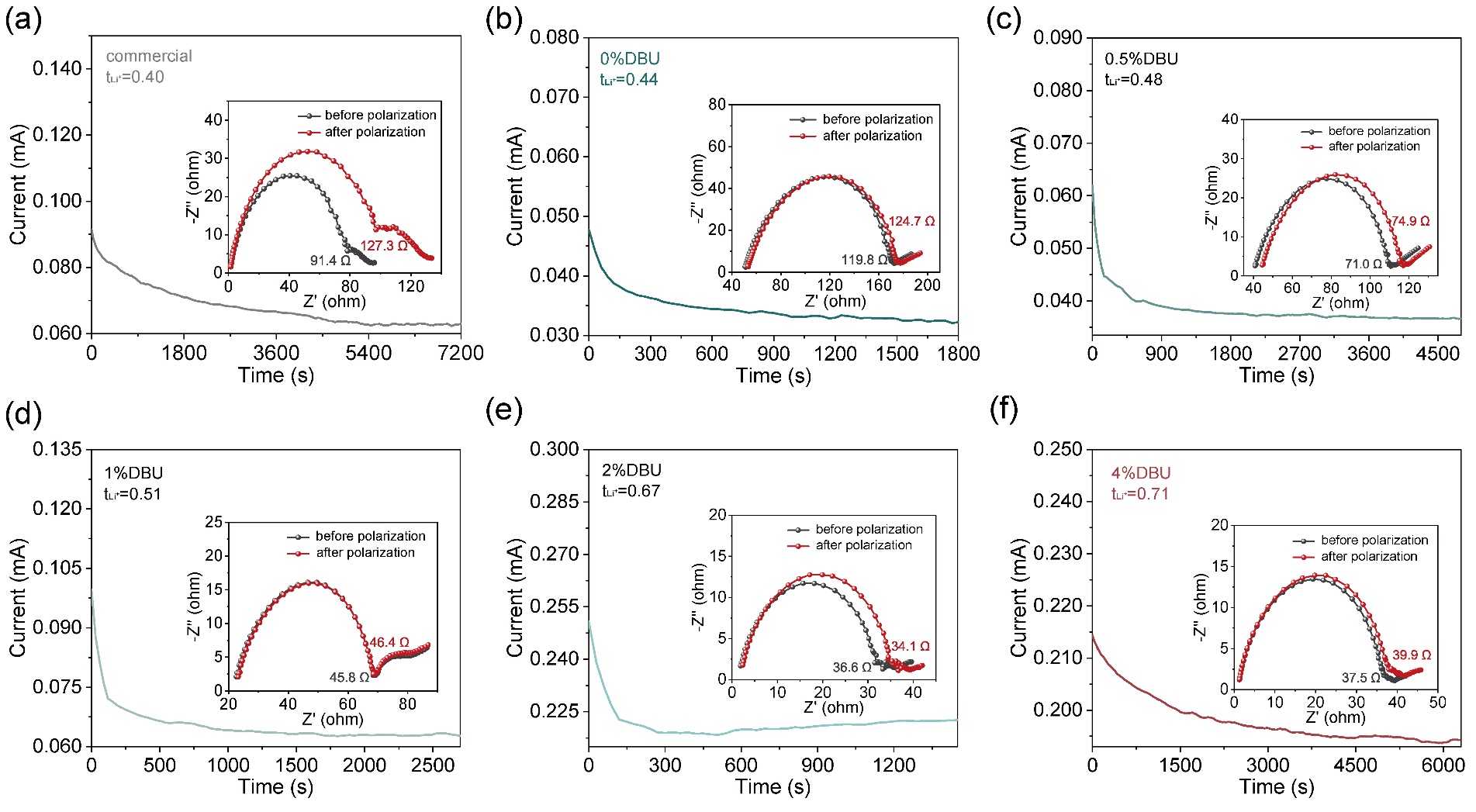


**Figure S11.** Testing of Li^+^ transference number for the (a) commercial electrolyte and DOL-based electrolytes with different DBU contents: (b) 0% DBU, (c) 0.5% DBU, (d) 1% DBU, (e) 2% DBU, and (f) 4% DBU.


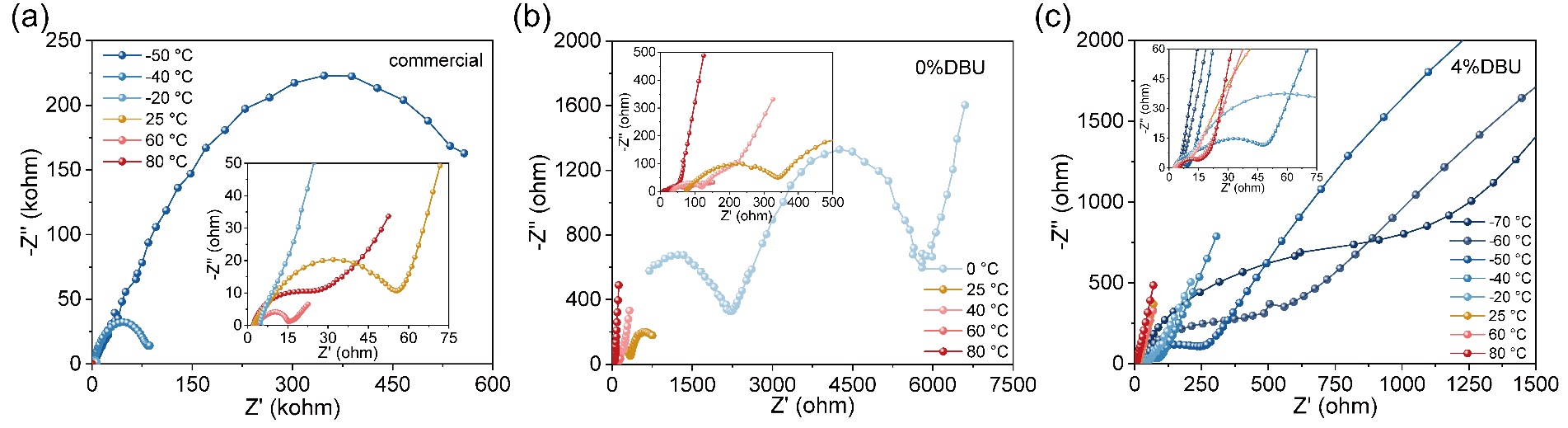


**Figure S12.** The Electrochemical Impedance Spectroscopy (EIS) of graphite half cells with the (a) commercial, (b) 0%DBU, and (c) 4%DBU electrolytes at different temperatures.


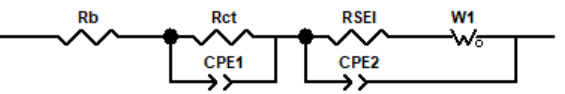


**Figure S13.** Equivalent circuit diagram for the EIS analysis.


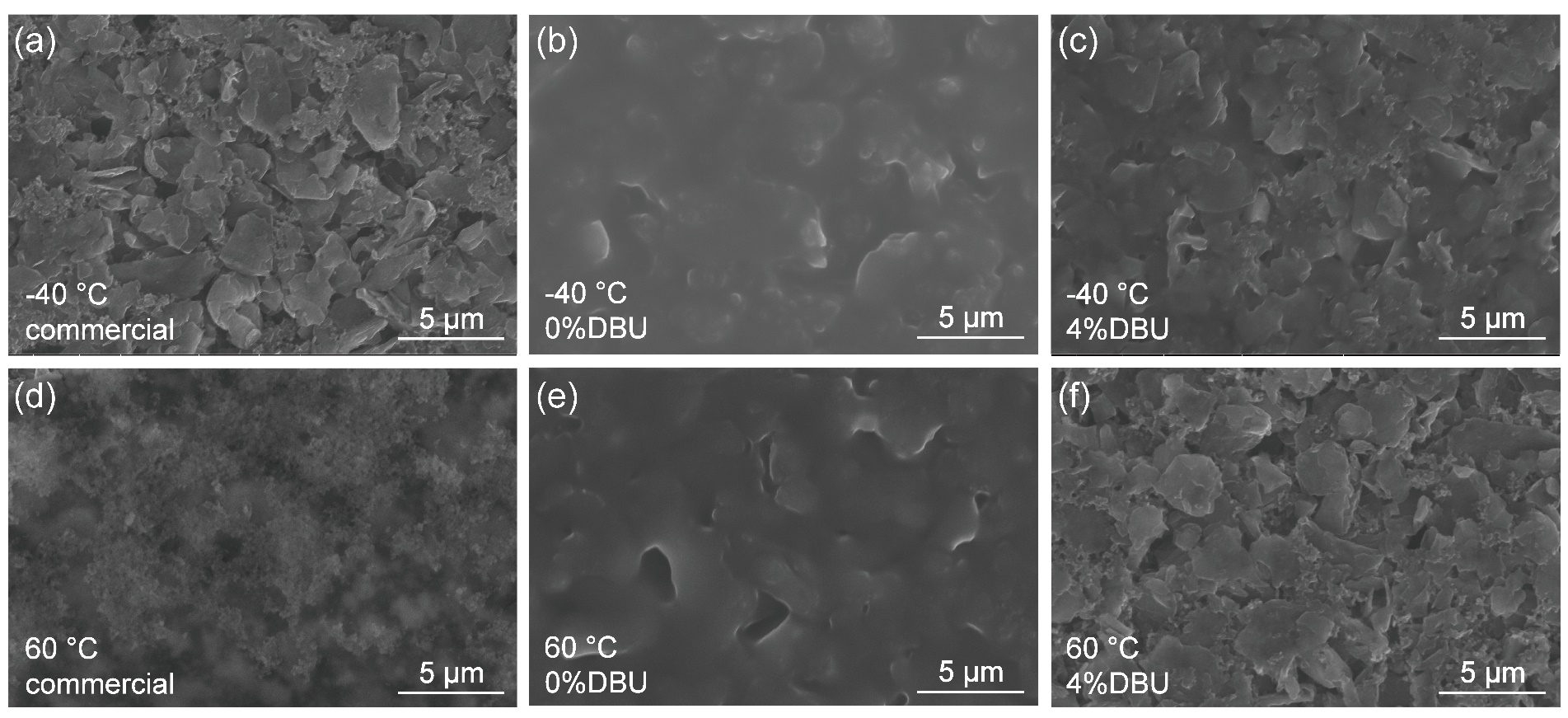


**Figure S14.** SEM surfaces of graphite electrodes after electrochemical testing at -40 °C in the (a) commercial, (b) 0%DBU, and (c) 4%DBU electrolytes, and after electrochemical testing at 60 °C in the (d) commercial, (e) 0%DBU, and (f) 4%DBU electrolytes.


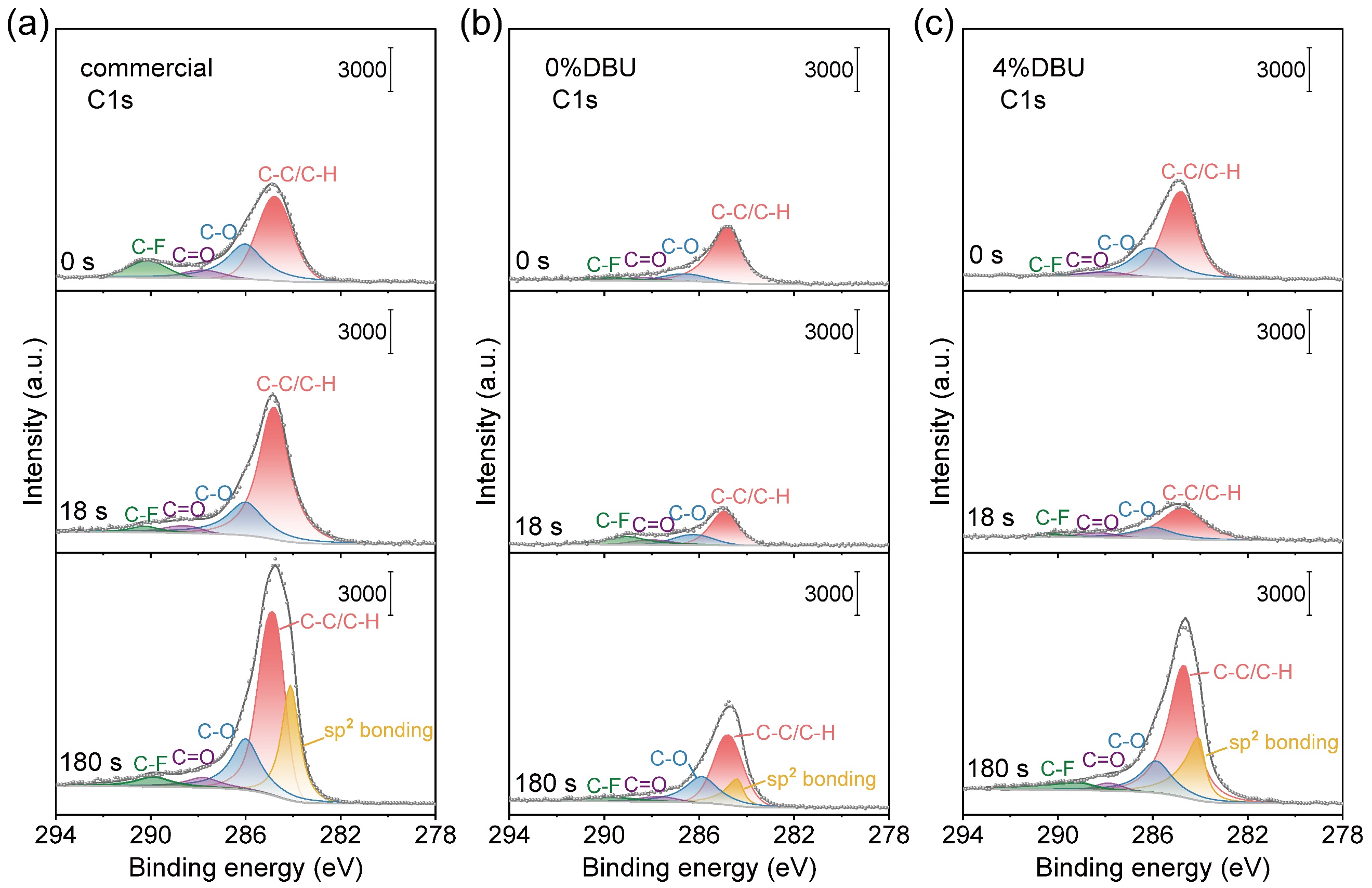


**Figure S15.** XPS C1s spectra of the SEI of graphite anodes after deintercalation of Li^+^ at room temperature in the (a) commercial, (b) 0%DBU and (c) 4%DBU electrolytes.


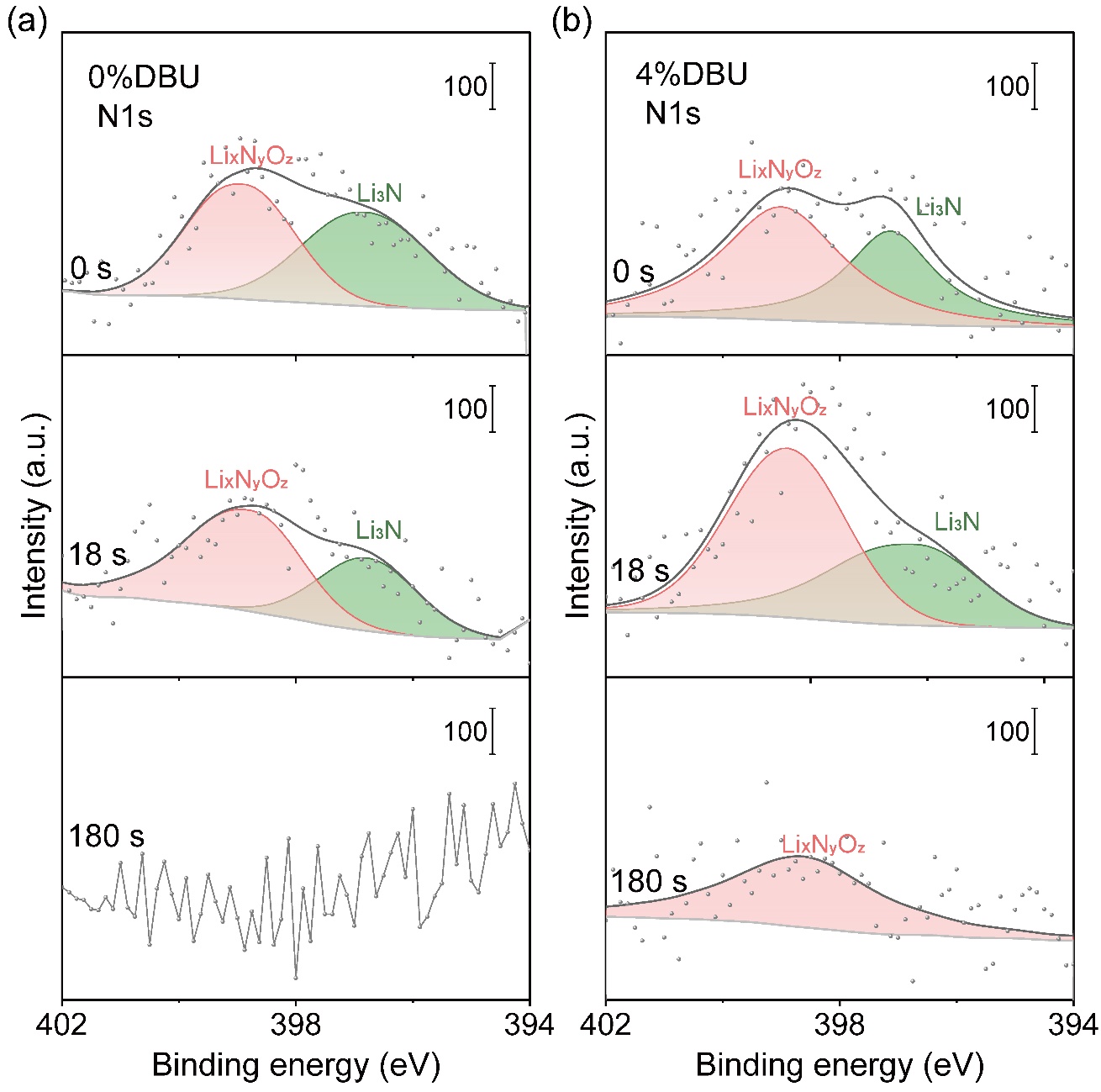


**Figure S16.** XPS N1s spectra of the SEI of graphite anodes after de-intercalation of Li^+^ at room temperature in the (a) 0%DBU and (b) 4%DBU electrolytes.


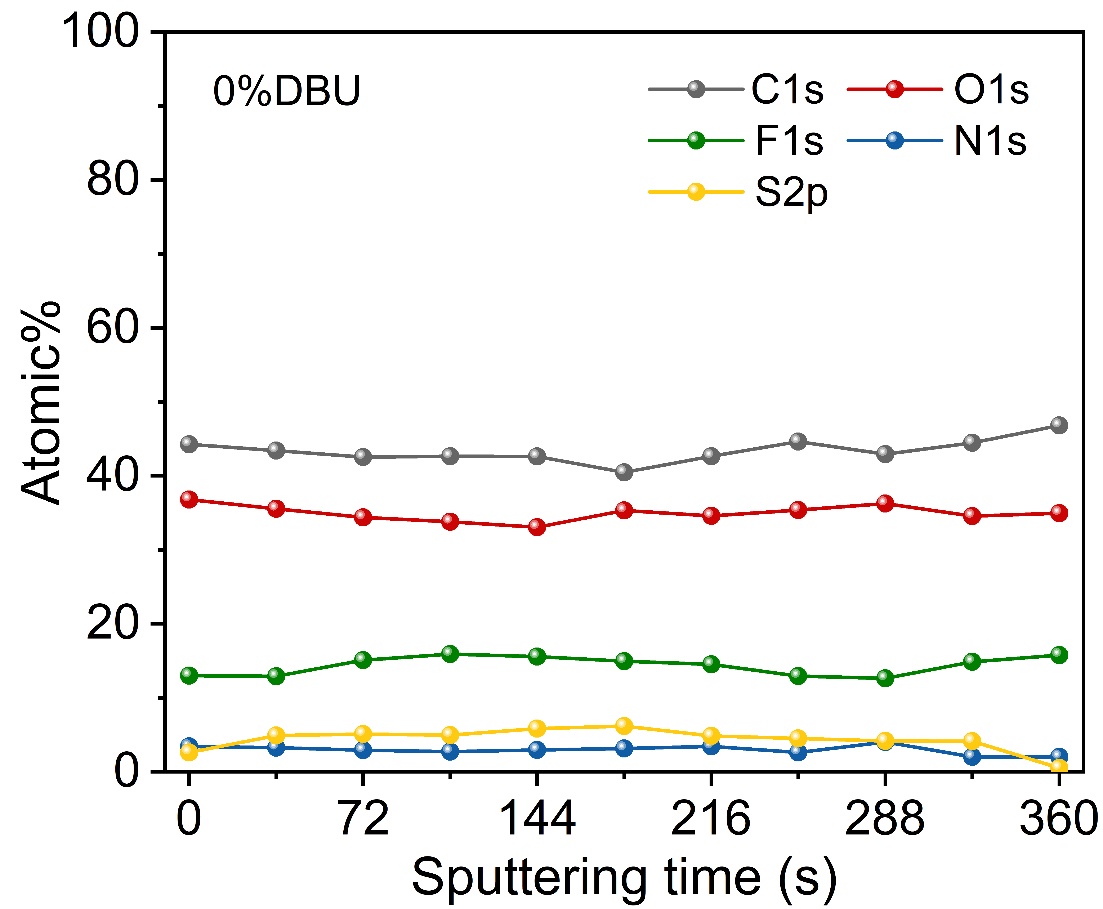


**Figure S17.** XPS general spectrum of graphite electrodes in the 0%DBU electrolyte system (uncleaned surface polymer electrolyte): the elemental percentages remained almost constant after 360 seconds of Ar^+^ sputtering, indicating that the polymer film was not penetrated.


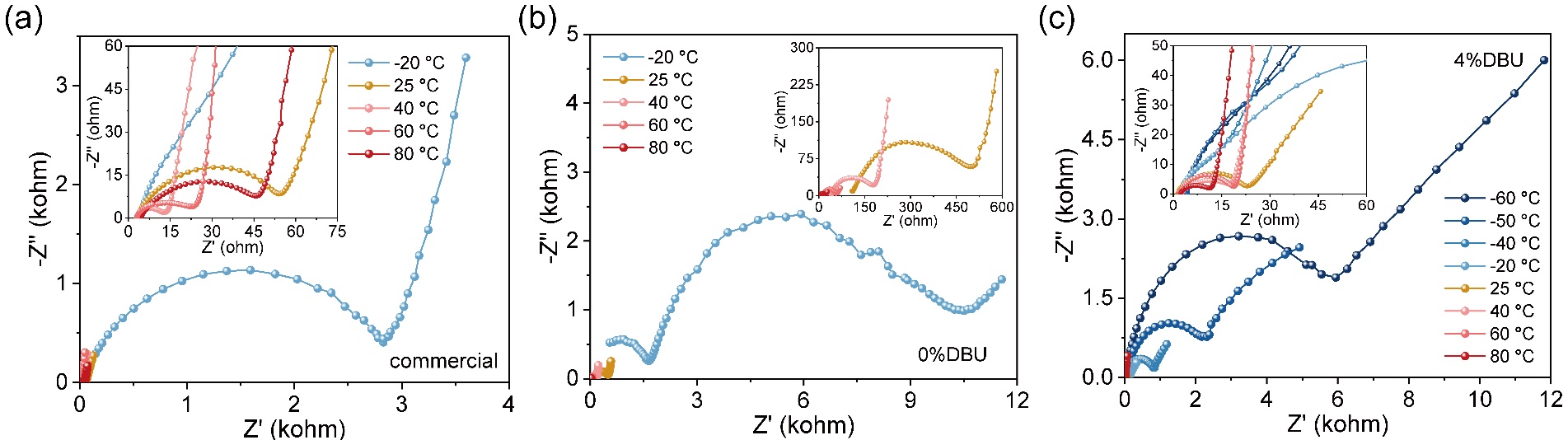


**Figure S18.**  The EIS of LFP half cells in the (a) commercial, (b) 0%DBU, and (c) 4%DBU electrolytes at different temperatures.


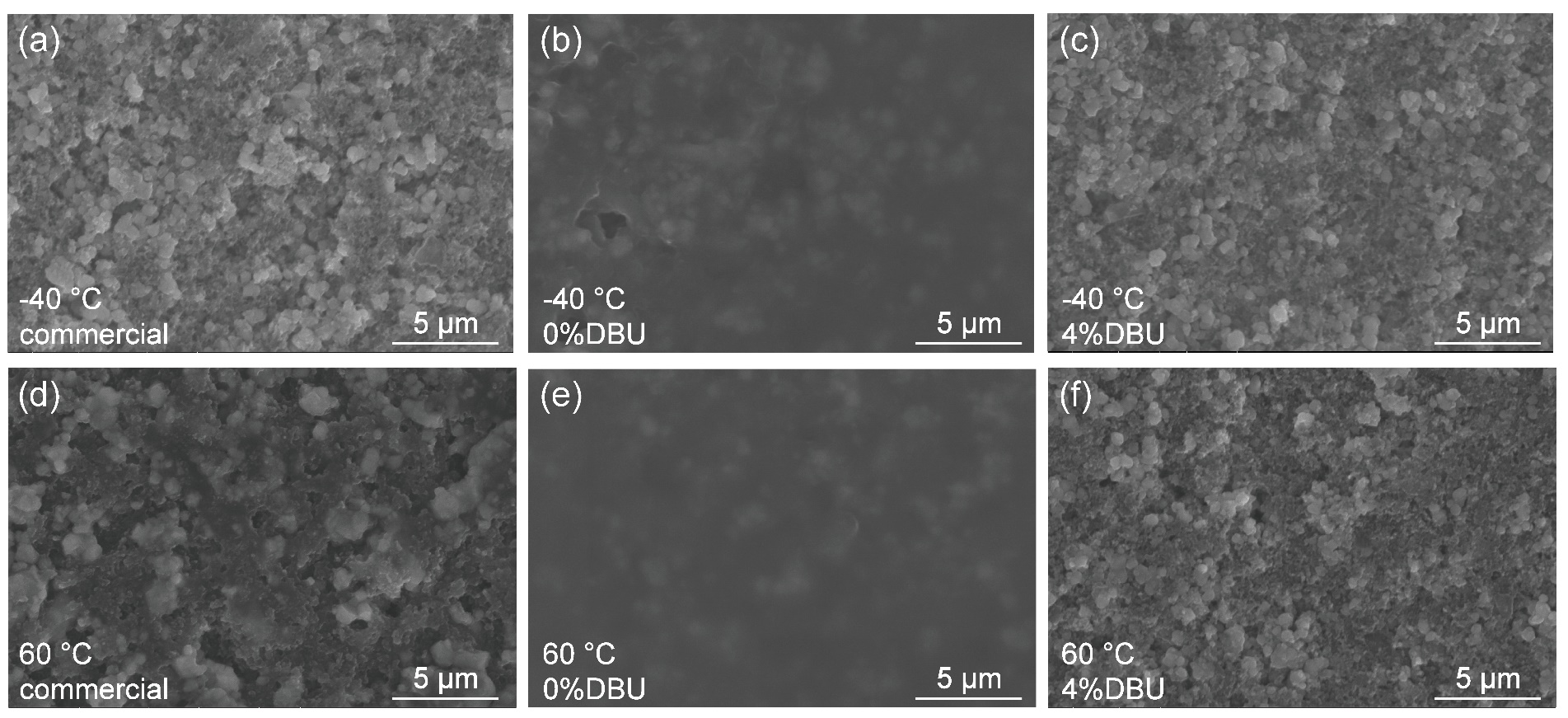


**Figure S19.** SEM images of LFP electrodes after electrochemical testing at -40 °C in the (a) commercial, (b) 0%DBU, and (c) 4%DBU electrolytes, and after electrochemical testing at 60 °C in the (d) commercial, (e) 0%DBU, and (f) 4%DBU electrolytes.


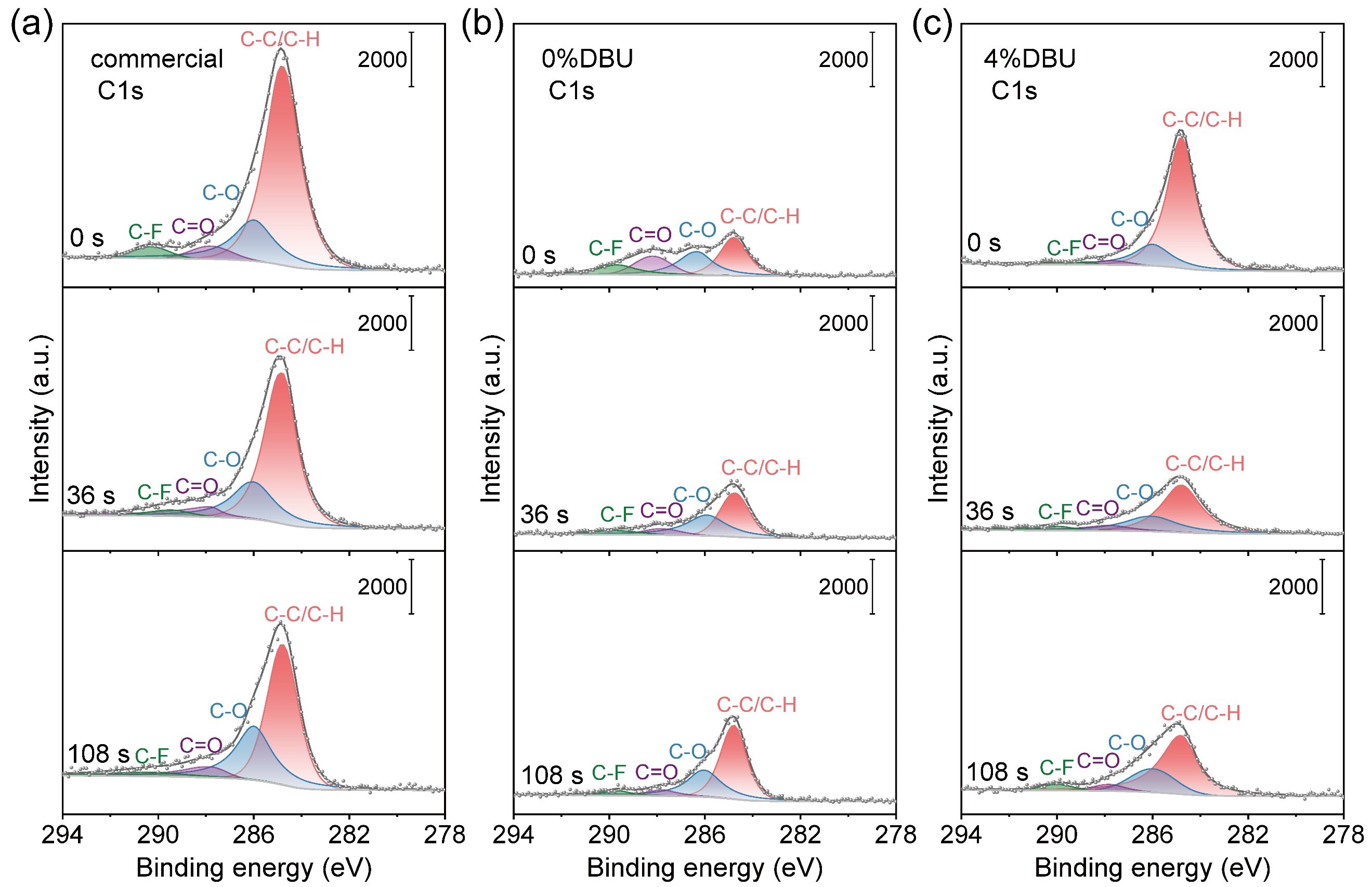


**Figure S20.** XPS C1s spectra of the CEI of graphite anodes after de-intercalation of Li^+^ at room temperature in the (a) commercial, (b) 0%DBU, and (c) 4%DBU electrolytes.


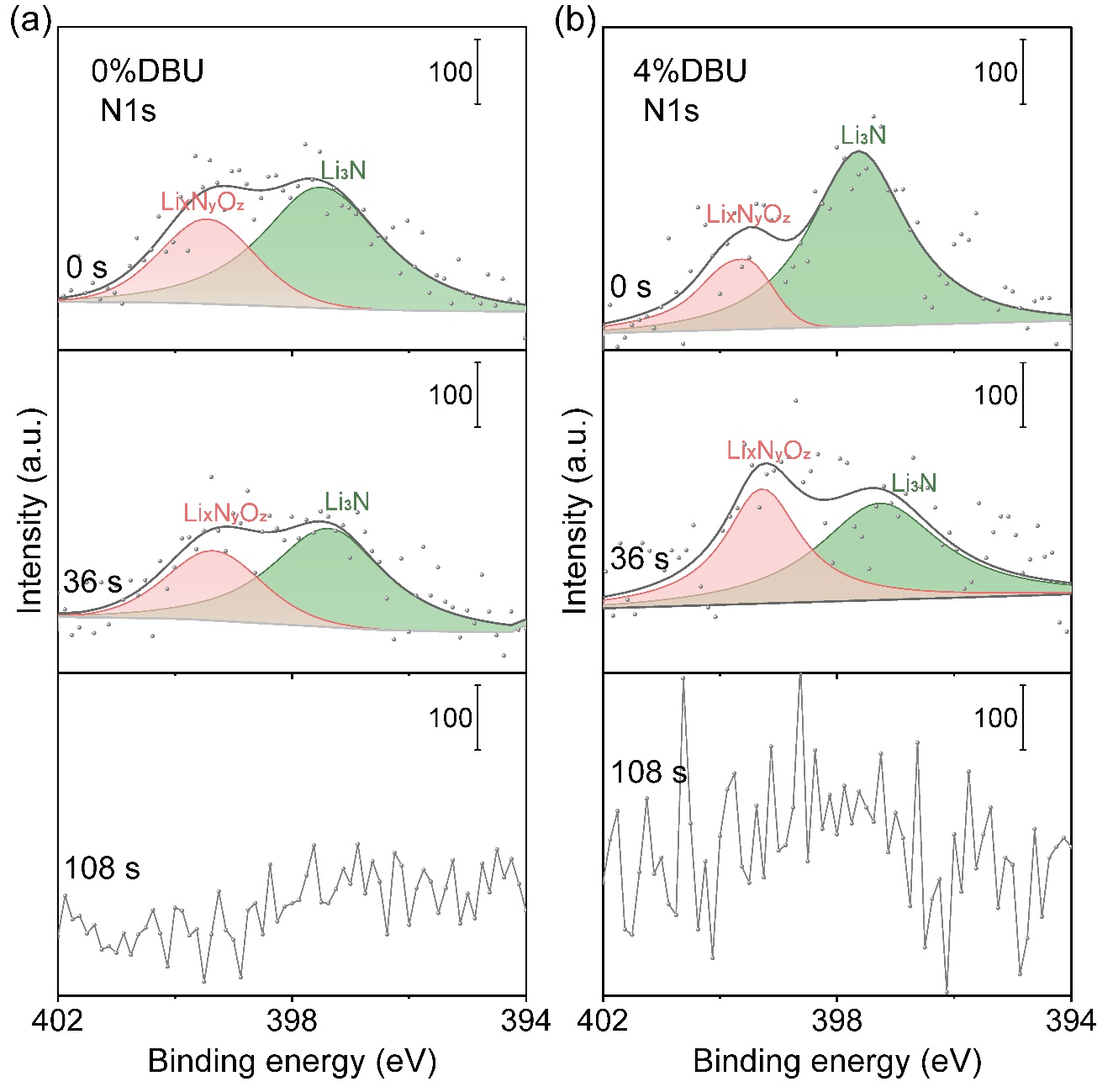


**Figure S21.** XPS N1s spectra of the CEI of graphite anodes after de-intercalation of Li^+^ at room temperature in the (a) 0%DBU and (b) 4%DBU electrolytes.


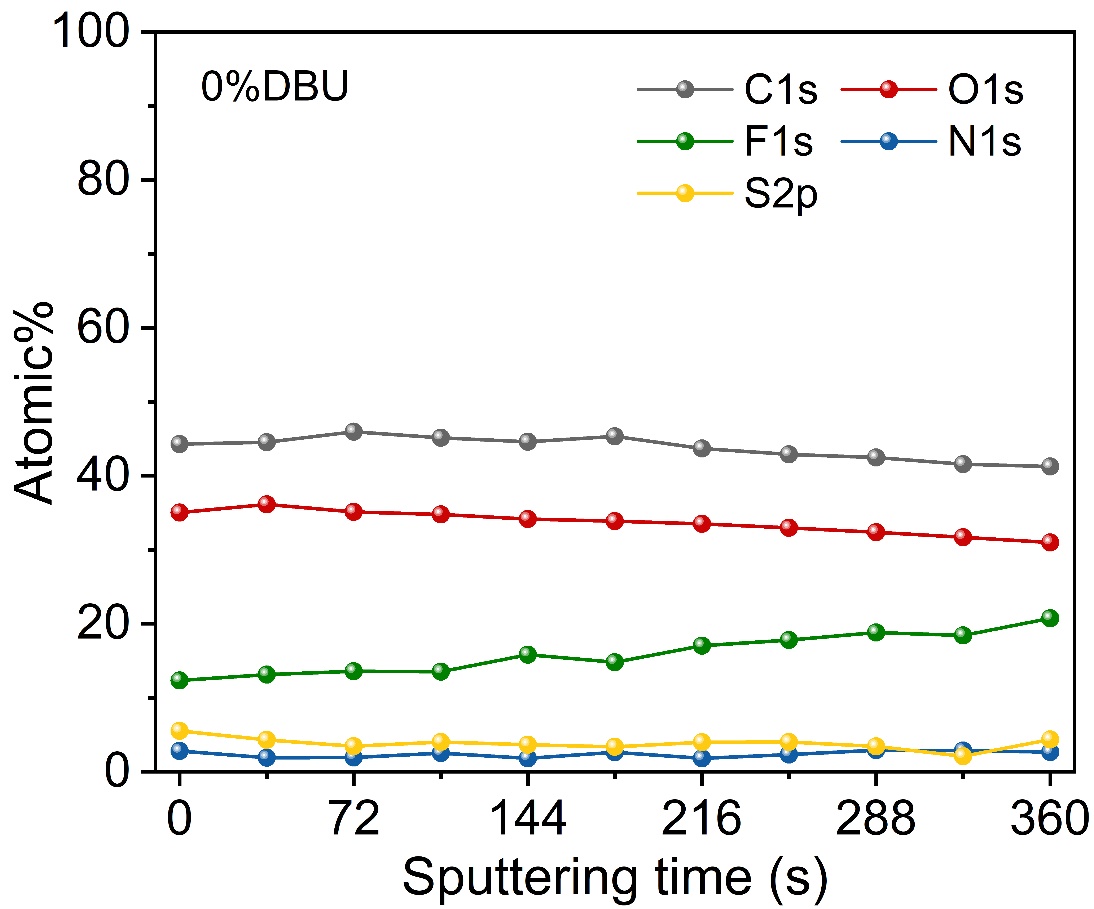


**Figure S22.** XPS general spectrum of LFP electrodes in the 0%DBU electrolyte (uncleaned surface polymer electrolyte): the elemental percentages remained almost constant after 360 s of Ar^+^ sputtering, indicating that the polymer film was not penetrated.


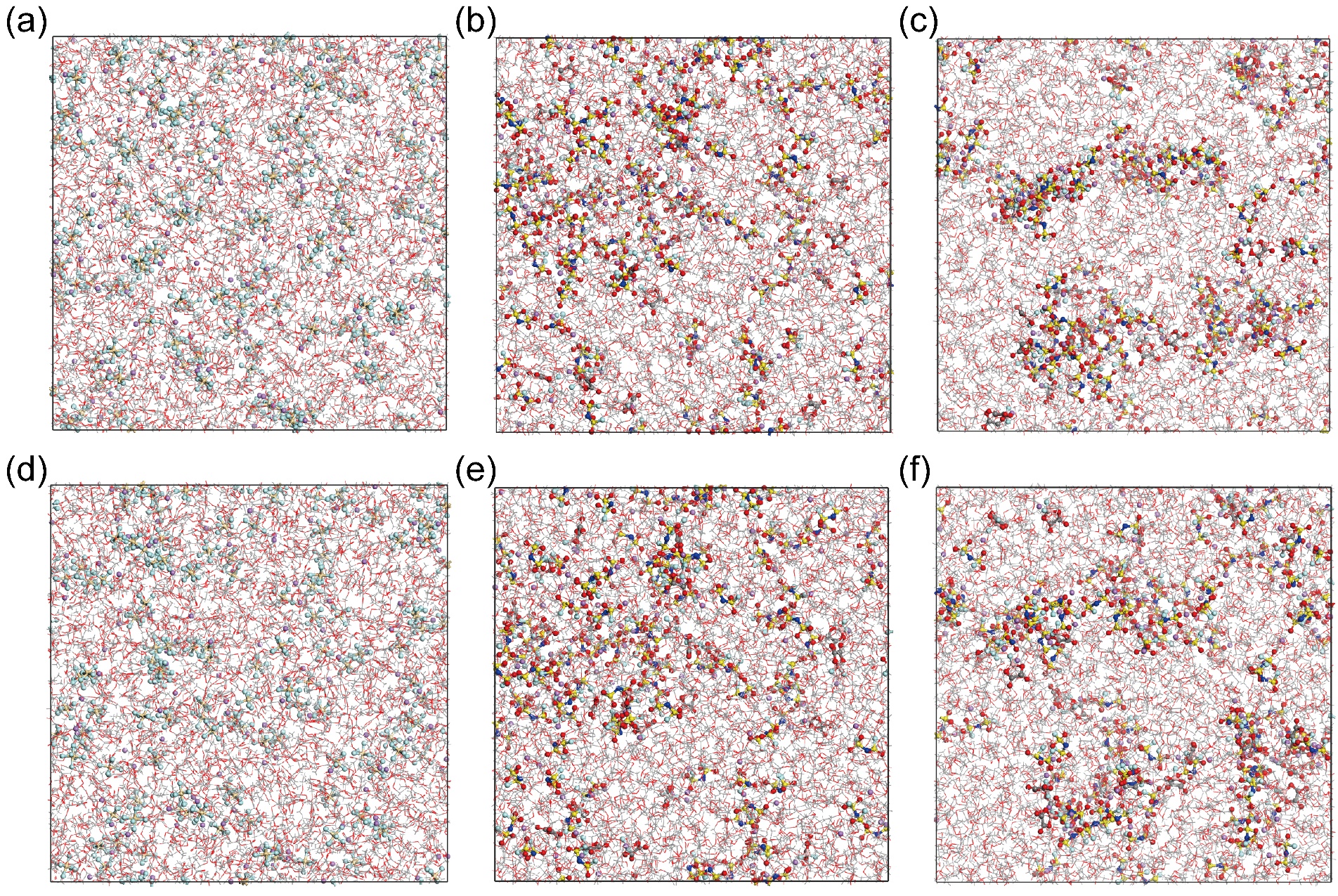


**Figure S23.** MD simulation snapshots of the (a) commercial, (b) 0%DBU, and (c) 4%DBU electrolytes at -60 °C and the (d) commercial, (e) 0%DBU, and (f) 4%DBU electrolytes at 60 °C.


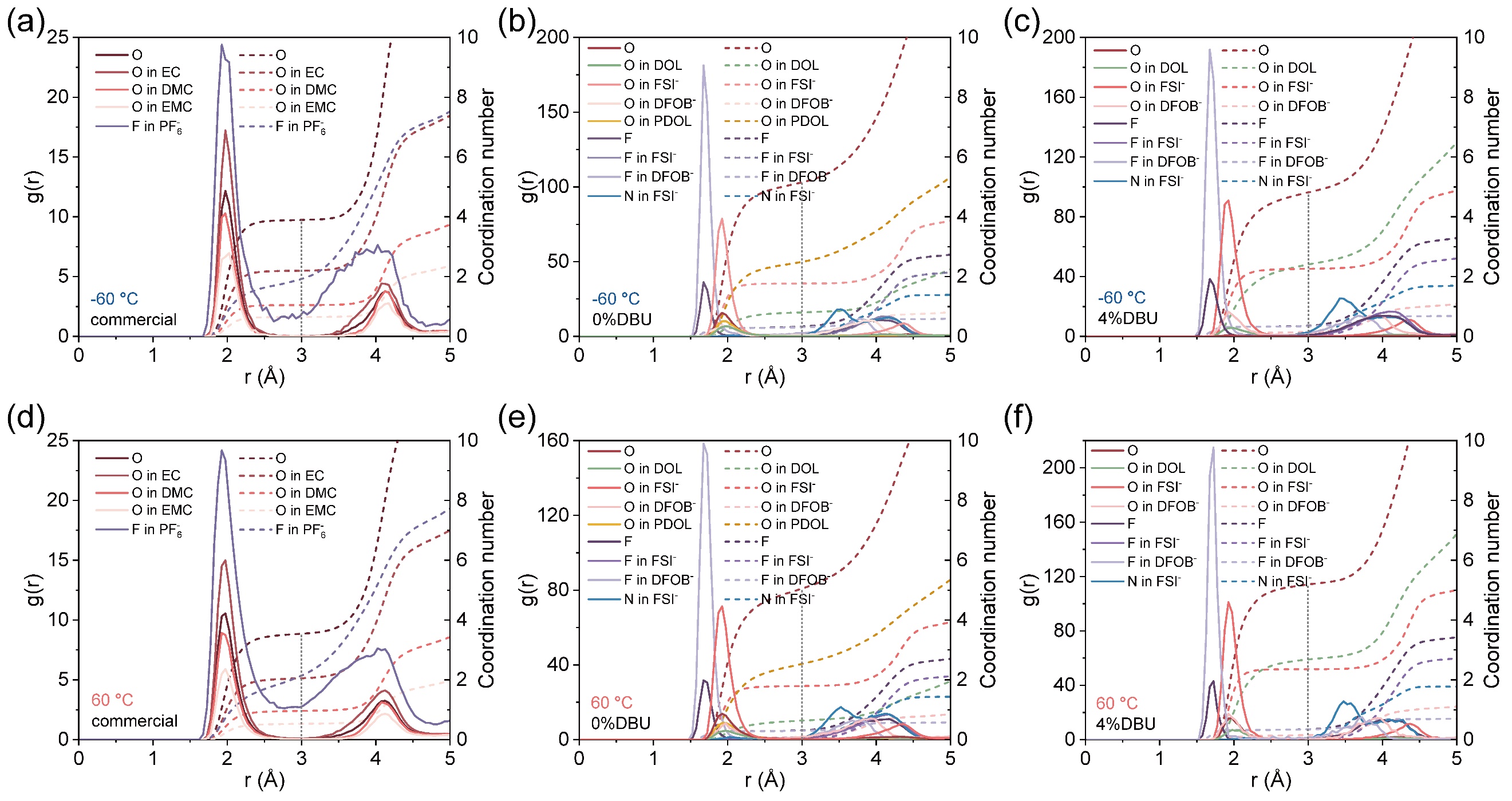


**Figure S24.** RDFs and CNs of the (a) commercial, (b) 0%DBU, and (c) 4%DBU electrolytes at -60 °C, and the (d) commercial, (e) 0%DBU, and (f) 4%DBU electrolytes at 60 °C.


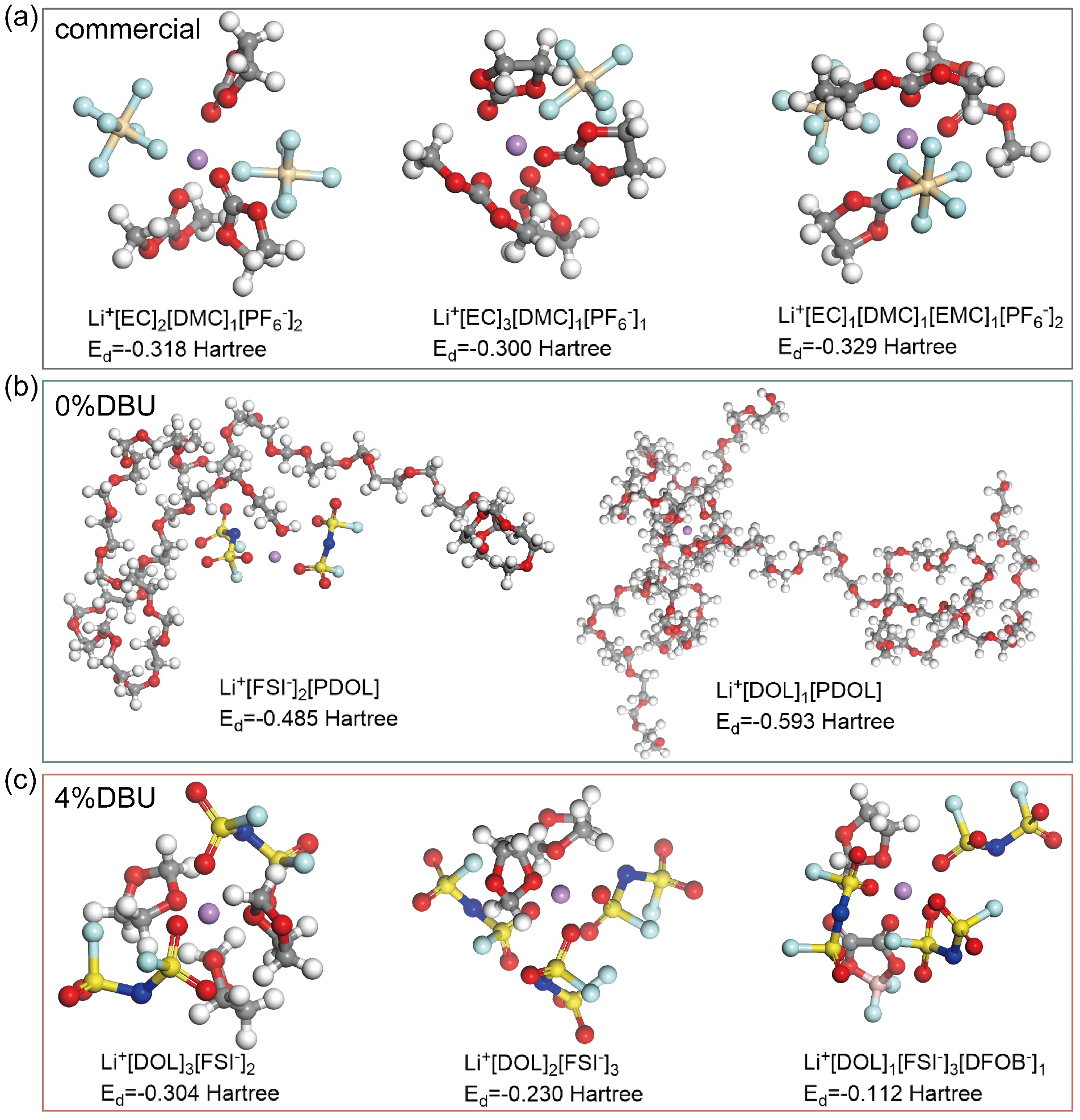


**Figure S25.** Other representative solvation structures for the (a) commercial, (b) 0%DBU, and (c) 4%DBU electrolytes (no significant difference at -60, 25, 60 °C).


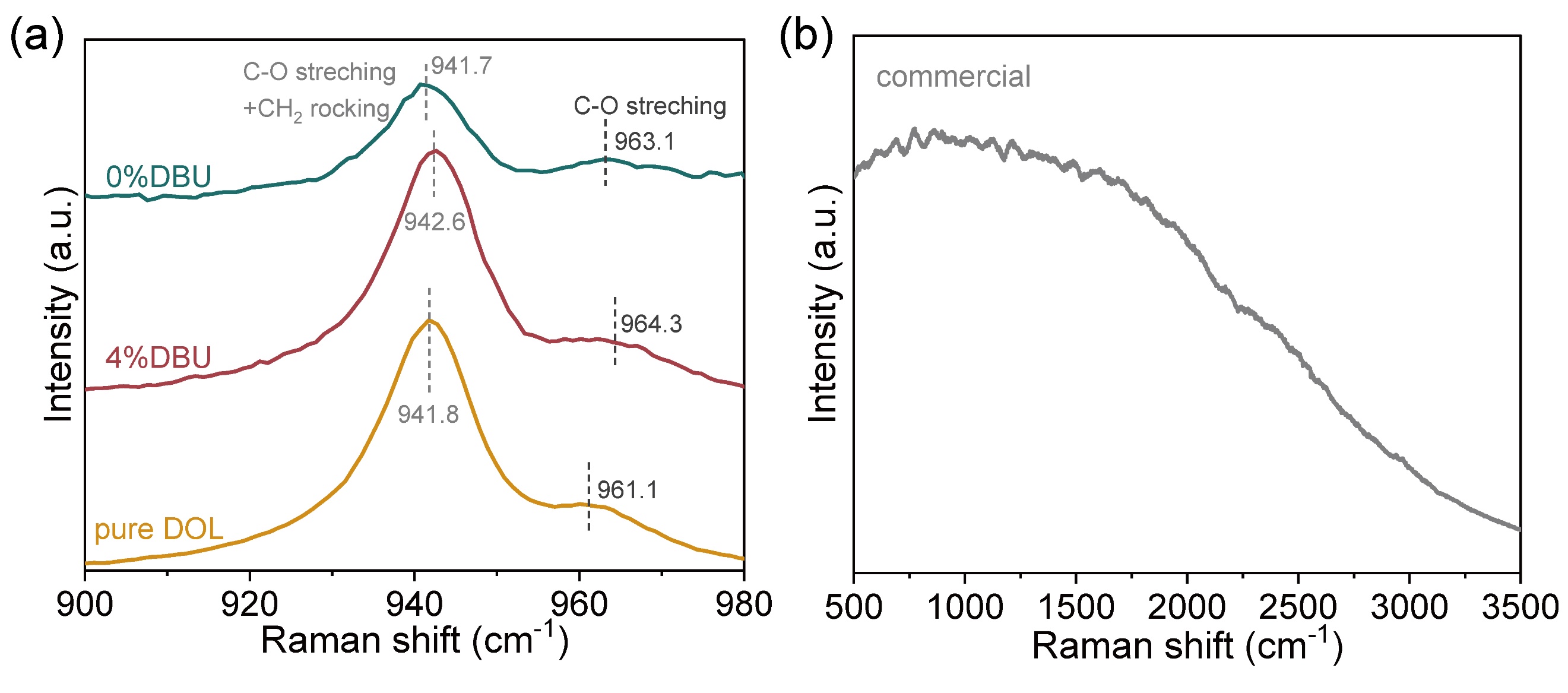


**Figure S26.** (a) Localized magnification of Raman spectra of pure DOL, the 0%DBU and 4%DBU electrolytes; (b) Raman spectrum of the commercial electrolyte. Fluorescence from commercial electrolyte components severely hampers the ability to examine them using a wavelength of 633 nm Raman spectroscopy. ^[9]^


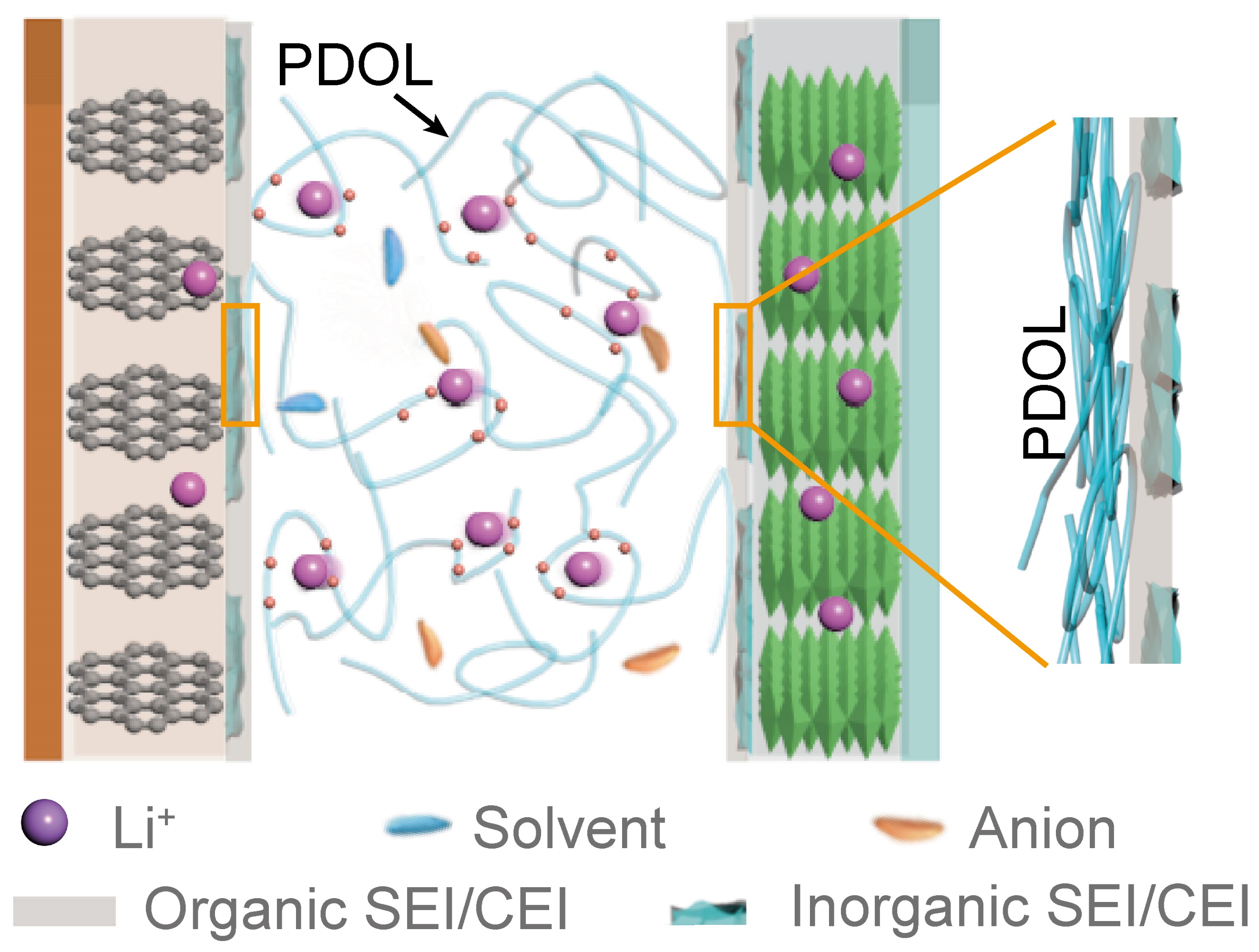


**Figure S27.** Localized enlarged schematic of the 0%DBU electrolyte-electrode interfaces.


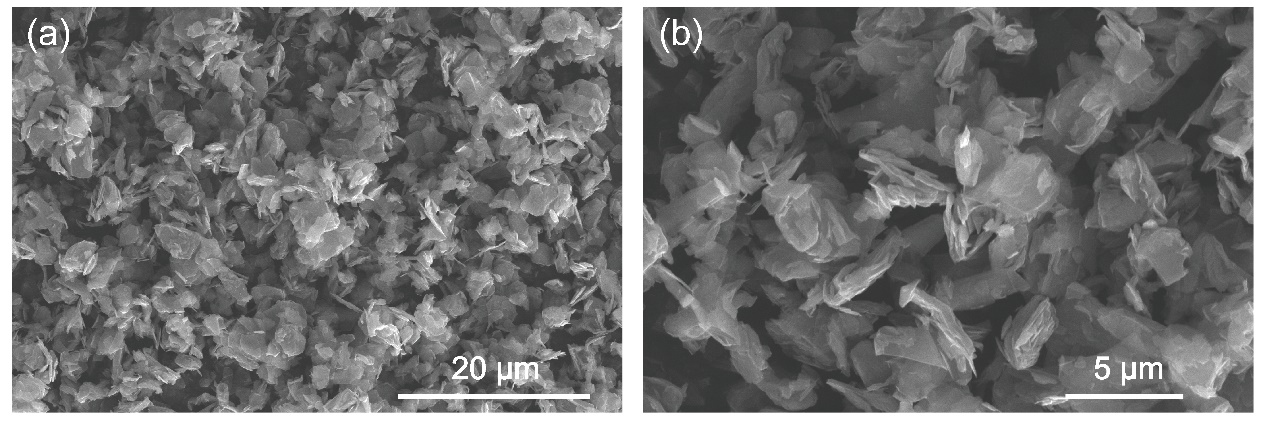


**Figure S28.** SEM images of graphite powder at different magnifications.


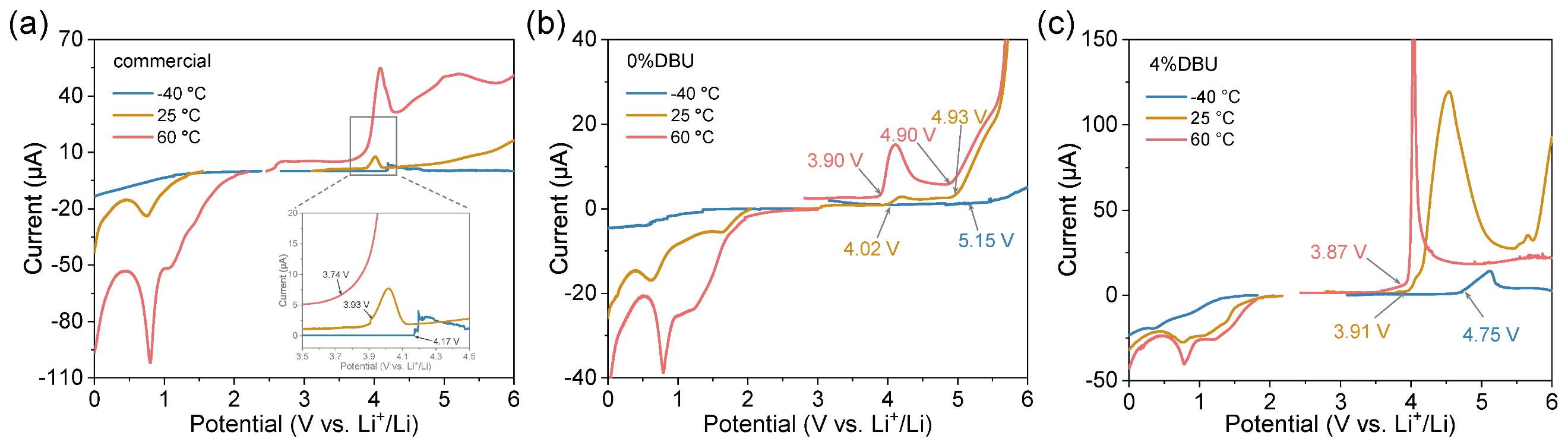


**Figure S29.** The LSV curves of the (a) commercial, (b) 0%DBU and (c) 4%DBU electrolytes at -40, 25 and 60 °C.


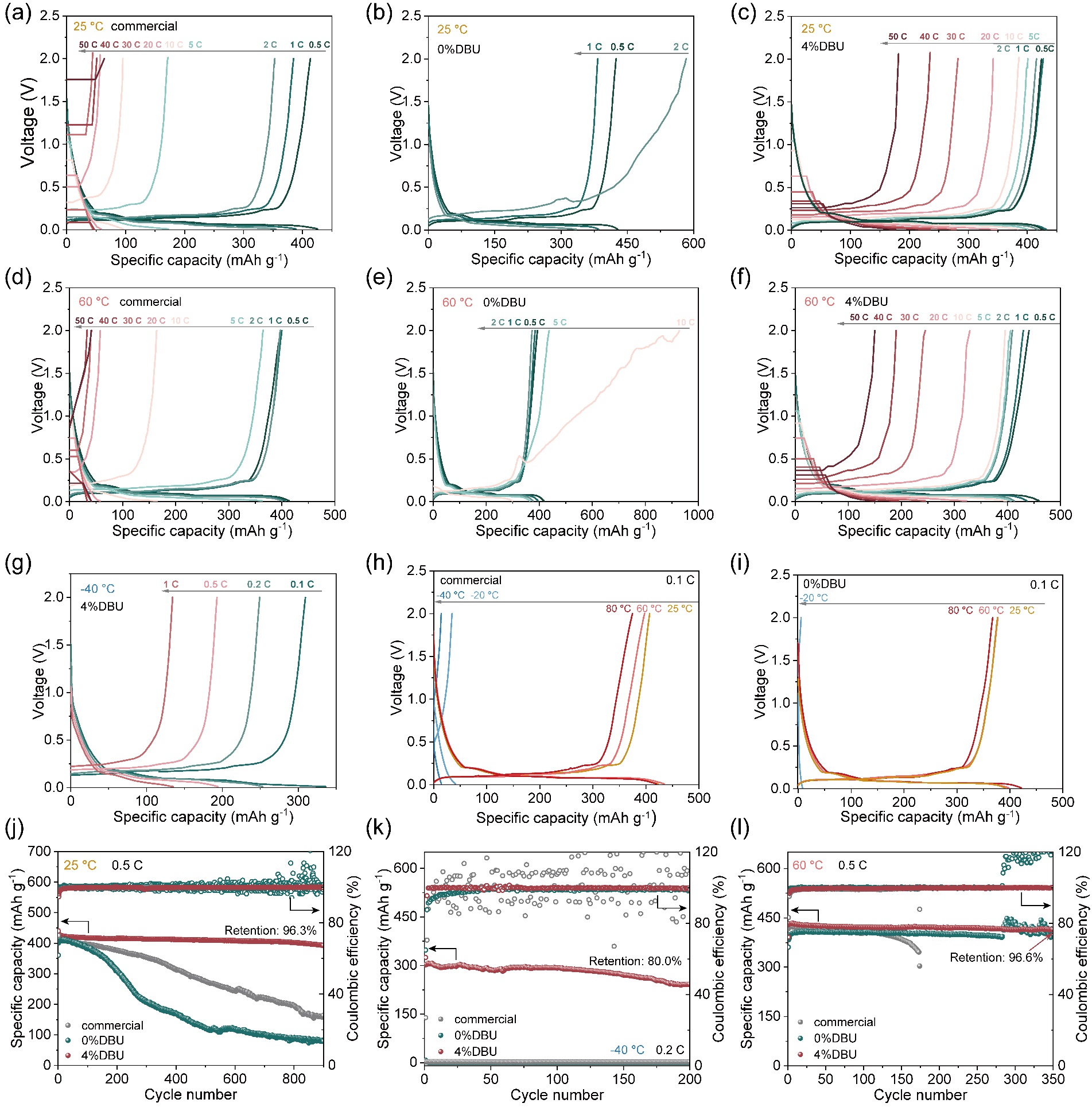


**Figure S30.** Electrochemical performance of graphite||Li half cells: Specific capacity-voltage curves at 25 °C in the (a) commercial, (b) 0%DBU, and (c) 4%DBU electrolytes, at 60 °C in the (d) commercial, (e) 0%DBU, and (f) 4%DBU electrolytes, and (g) at -40 °C in the 4%DBU electrolyte; Specific capacity-voltage curves at different temperatures in the (h) commercial and (i) 0%DBU electrolytes at 0.1 C; Cycling performance at (j) room temperature at 0.5 C, (k) -40 °C at 0.2 C, and (l) 60 °C at 0.5 C in the three electrolytes.


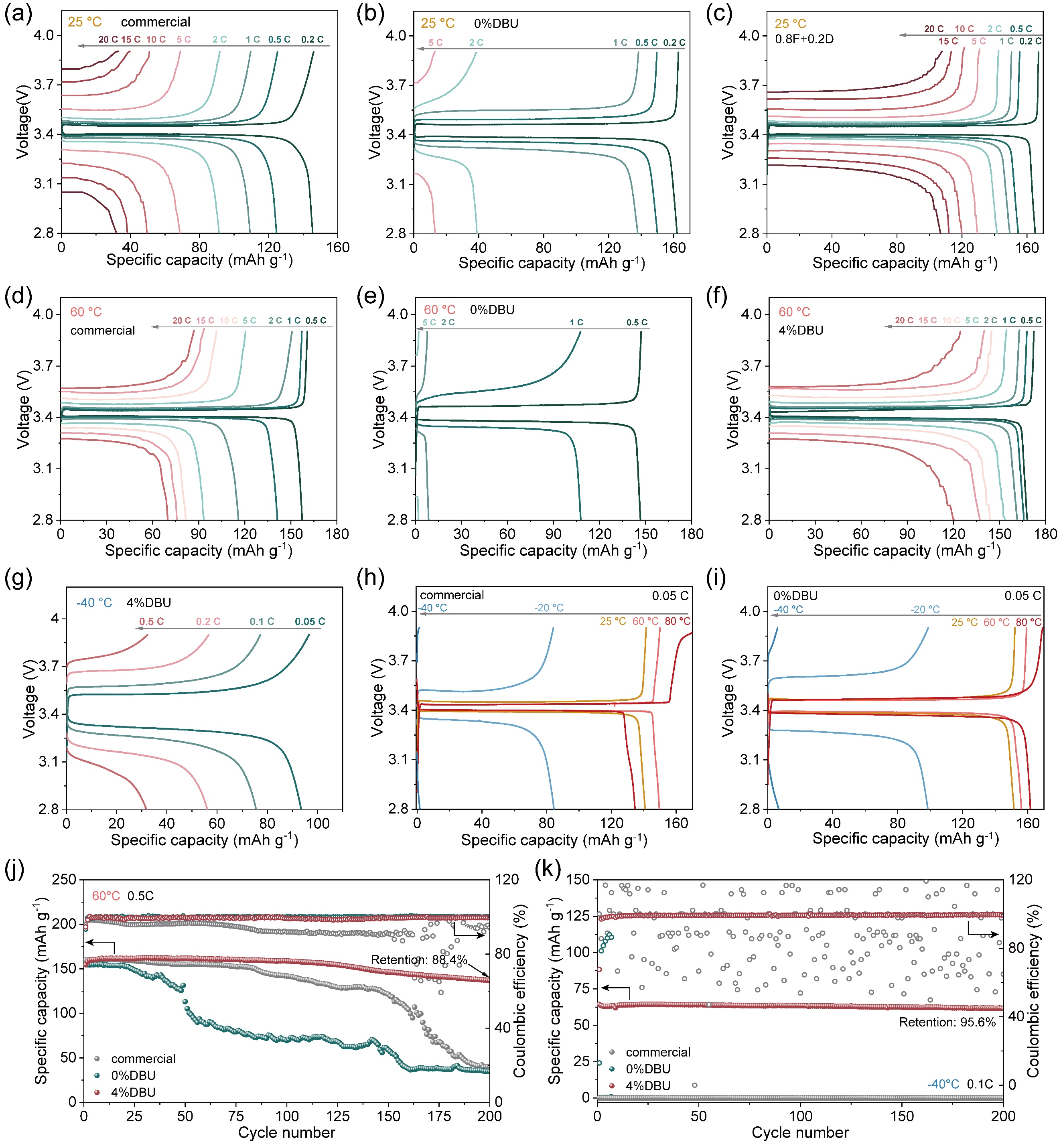


**Figure S31.** Electrochemical performance of LFP||Li half cells: Specific capacity-voltage curves of cells at 25 °C in the (a) commercial, (b) 0%DBU, and (c) 4%DBU electrolytes, at 60 °C in the (d) commercial, (e) 0%DBU, and (f) 4%DBU electrolytes, and at -40 °C in the 4%DBU electrolyte; Specific capacity-voltage curves at different temperatures in the (h) commercial and (i) 0%DBU electrolytes at 0.1 C; Cycling performance of cells at (j) -40 °C at 0.2 C, and (k) 60 °C at 0.5 C in the three electrolytes.


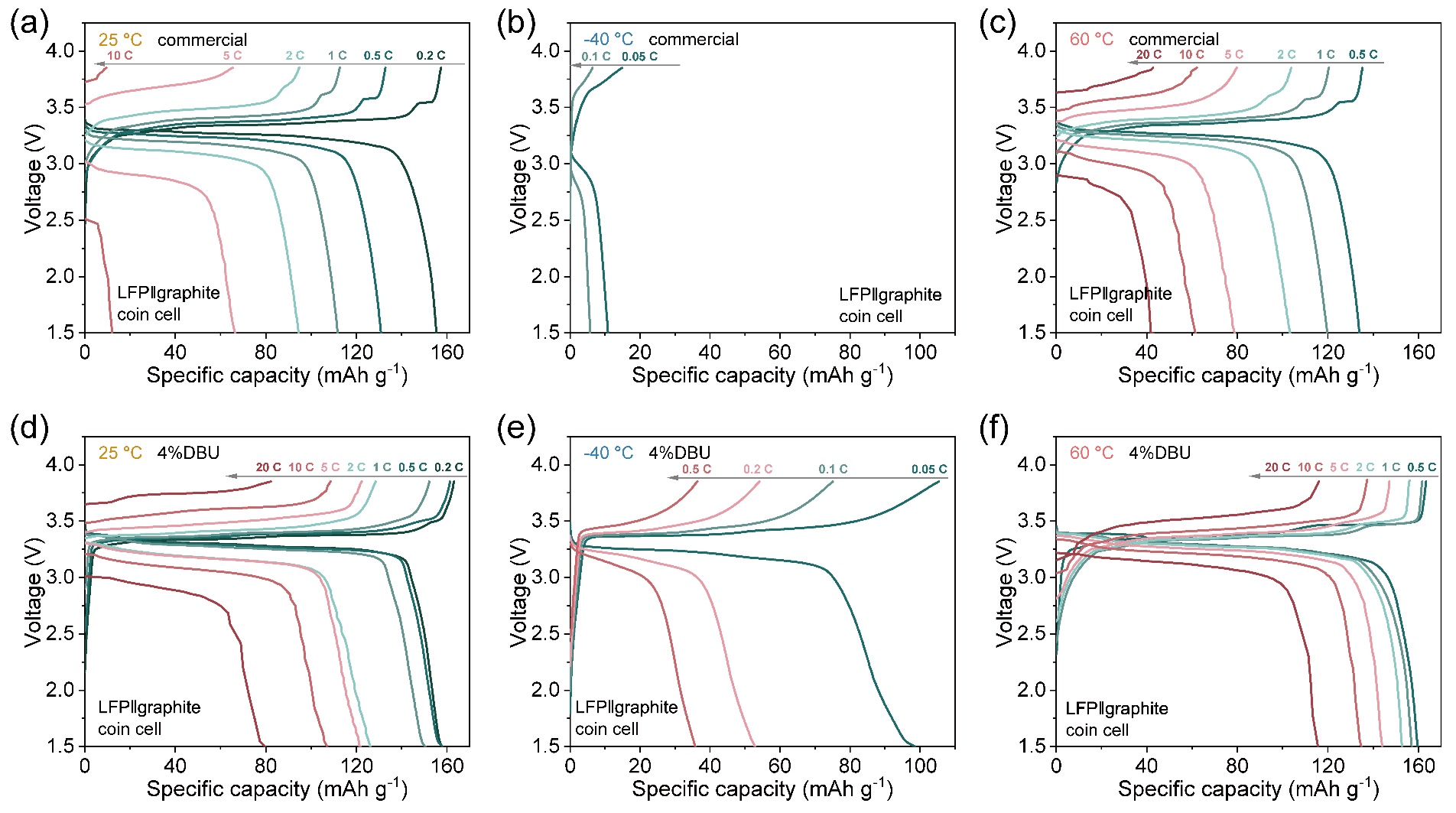


**Figure S32.** Electrochemical performance of LFP||graphite coin cells: Specific capacity-voltage curves in the commercial electrolyte at (a) 25, (b) -40 and (c) 60 °C and the 4%DBU electrolyte at (d) 25, (e) -40, and (f) 60 °C.


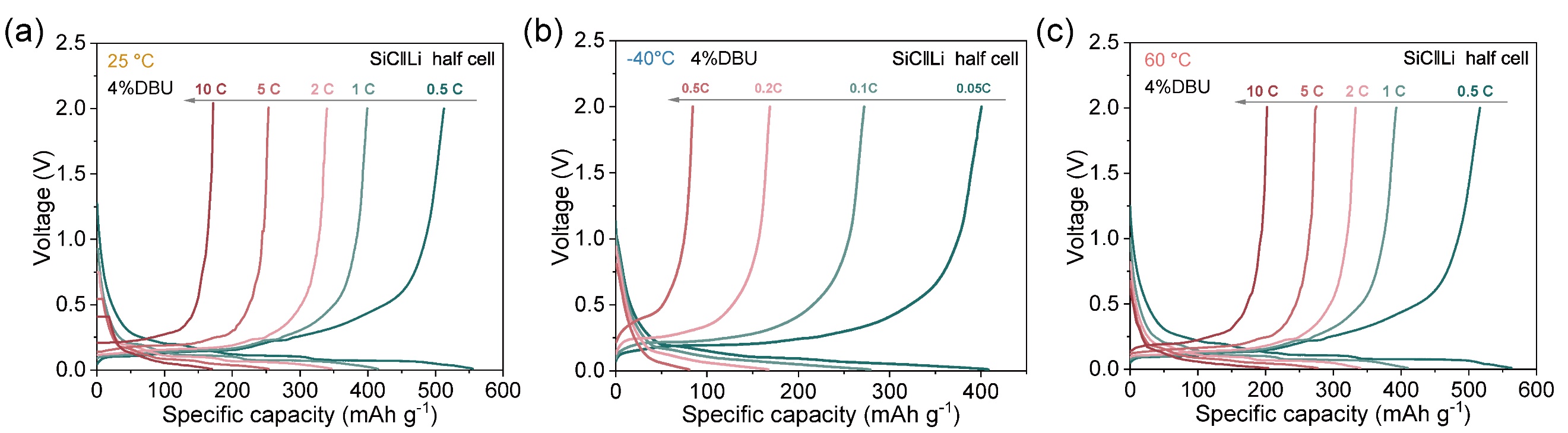


**Figure S33.** Electrochemical performance of SiC||Li half cells: Specific capacity-voltage curves in the 4%DBU electrolyte at (a) 25, (b) -40 and (c) 60 °C.

**Table S1.** Comparison of different physical parameters of various organic solvents. ^[10–12]^

| Solvents | Melting point (°C) | Boiling point (°C) | Dielectric constants | Donor number | Li^+^-solvent binding energy(eV) |
| --- | --- | --- | --- | --- | --- |
| Ethylene carbonate (EC) | 36.4 | 248 | 89.8 | 16.4 | -2.239 |
| Dimethyl carbonate (DMC) | 4 | 90 | 3.1 | 15.2 | -1.957 |
| Diethyl carbonate (DEC) | -43 | 126 | 2.8 | 16.0 | -2.089 |
| Ethyl methyl carbonate (EMC) | -53 | 110 | 3.0 | 17.2 | -2.025 |
| Propylene carbonate (PC) | -49 | 242 | 64.6 | 15.1 | -2.313 |
| Methyl acetate (MA) | -98 | 57 | 7.3 | 16.5 | -2.063 |
| Ethyl acetate (EA) | -84 | 77 | 6.4 | 17.1 | -2.133 |
| Methyl propanoate (MP) | -8 | 80 | 6.1 | 11.0 | -2.092 |
| Acetonitrile (AN) | -45 | 82 | 36.0 | 14.1 | -1.986 |
| Trimethyl phosphate (TMP) | -46 | 197 | 20.6 | 23.0 | -2.601 |
| Dimethyl sulfoxide (DMSO) | 18 | 189 | 46.4 | 29.8 | -2.656 |
| Tetrahydrofuran (THF) | -108 | 65 | 7.6 | 21.0 | -1.995 |
| Dimethoxyethane (DME) | -58 | 85 | 7.2 | 20.0 | -2.817 |
| 1,3-Dioxolane (DOL) | -95 | 74 | 7.3 | 21.2 | -1.713 |

**Table S2.** Comparison of the fundamental physical properties of various organic bases.^[13–16]^

| Base | CAS | pK_a_  (in water) | pK_a_  (in AN) | ESP_min_  (kcal mol^-1^) | ESP_max_  (kcal mol^-1^) | Melting point (°C) | Boiling point (°C) |
| --- | --- | --- | --- | --- | --- | --- | --- |
| TMG | 80-70-6 | 13.0 | 23.3 | -44.49486 | 17.30066 | -30 | 163 |
| Et3N | 121-44-8 | 10.8 | 18.8 | -29.20539 | 10.84726 | -115 | 90 |
| DIPEA | 7087-68-5 | 11.4 | N/A | -27.89629 | 10.01053 | -46 | 127 |
| 2,6-lutidine | 108-48-5 | N/A | N/A | -36.22478 | 1822577 | -6 | 144 |
| 2,6-di-tert-butylpyridine | 585-48-8 | N/A | N/A | -21.23862 | 17.49581 | 2 | 209 |
| DBN | 3001-72-7 | N/A | 23.4 | -46.78759 | 15.25578 | N/A | 238 |
| DBU | 6674-22-2 | 11.5 | 23.9 | -45.47519 | 17.63086 | -70 | 275 |

**Note:**

The following lists the full names of the reagents mentioned in the table:

1,1,3,3-Tetramethylguanidine (TMG)

Triethylamine (Et3N)

N,N-Diisopropylethylamine (DIPEA)

1,5-Diazabicyclo[4.3.0]non-5-ene (DBN )

**Table S3.** Comparison of LIBs employing graphite anodes for extreme temperature and high rate applications.

| Ref. | Journal | Electrolyte | Graphite\|\|Li half cell performance | Full cell performance  (capacity retention relative to room temperature at a low rate) |
| --- | --- | --- | --- | --- |
| [17] | **Adv. Mater**. | 1 M LiFSI in DiFEC:MTFC: HFME=1:2:2 vol% | 215.5 mAh g^-1^, -20 °C, 0.1 C  ~300 mAh g^-1^, 25 °C, 0.5 C | NCM523\|\|graphite coin cell  50.9%, -40 °C, 0.1 C  58.5%, 25 °C, 4 C |
|  |  |  |  | NCM523\|\|graphite pouch cell  62%, -40 °C, 0.05 C |
| [18] | **Adv. Funct. Mater**. | 0.75 M LiFSI relative to TFMSA, TFMSA:TTE =7:3 vol% | 230 mAh g^-1^, -40 °C, 0.05 C  ~330 mAh g^-1^, 25 °C, 0.5 C  ~350 mAh g^-1^, 80 °C, 0.5 C | LiCoO_2_\|\|graphite pouch cell  50%, -40 °C, 0.05 C |
| [19] | **Adv. Energy Mater**. | LiFSI: PC: FB=1:5:7 mole% | 250 mAh g^-1^, 25 °C, 6 C  ~360 mAh g^-1^, 60 °C, 1 C  130 mAh g^-1^, -40 °C, 0.05 C | NCM811\|\|graphite coin cell  59.5%, -40 °C, 0.05 C (Discharge only)  101.2%, 60 °C, 0.05 C (Discharge only) |
| [20] | **Angew. Chemie Int. Ed**. | LiFSI: EMC: TTE =2:3.3:3.3 mole% | 307 mAh g^-1^, -20 °C, 0.33 C  150 mAh g^-1^, -20 °C, 0.8 C  ~330 mAh g^-1^, 25 °C, 3 C | NCM811\|\|graphite coin cell  54.5%, -40 °C, 0.1 C  54.0%, 25 °C, 4 C  ~70%, 50 °C, 0.33 C |
| [21] | **Nature** | 1 M LiTFSI in MDFA:MDFSA:TTE  =4:1:5 vol% | / | NCM811\|\|graphite coin cell  54%, -60 °C, 0.1 C  ~70%, 25 °C, 4 C  ~105%, 60 °C, 0.1 C |
| [22] | **ACS nano** | 1 M LiTFSI in FEC:CPME=3:7 vol% | 319 mAh g^-1^, -60 °C, 0.1 C (Charge only)  ~170 mAh g^-1^, 25 °C, 5 C | LiFePO_4_\|\|graphite pouch cell  31%, -60 °C, 0.1 C (Discharge only) |
| [23] | **Angew. Chemie** | 1 M LiTFSI in ETFA:FEC=7:3 vol% | 183 mAh g^-1^, -30 °C, 0.05 C  ~180 mAh g^-1^, 25 °C, 6 C | LiFePO_4_\|\|graphite coin cell  37%, -60 °C, 0.1 C (Discharge only) |
| **This work** | | 0.8 M LiFSI+0.2 M LiDFOB in DOL (with 4vol% DBU) | 310.5 mAh g^-1^, -40 °C, 0.1 C  141.7 mAh g^-1^, -40 °C, 1 C  181.6 mAh g^-1^, 25 °C, 50 C  155 mAh g^-1^, 60 °C, 50 C | LiFePO_4_\|\|graphite coin cell  28.5%, -60 °C, 0.05 C  62.4%/24.1%, -40 °C, 0.05 C/0.5 C  49.2%, 25 °C, 20 C  77.1%, 60 °C, 20 C |
|  |  |  |  | LiFePO_4_\|\|graphite pouch cell  60.4%, -40 °C, 0.1 C  75.6%, 60 °C, 0.5 C |

**Note:**

The default battery performance is rechargeable unless indicated otherwise in the table for individual charge or discharge performance.

In this work, the capacity retention of the LFP||graphite coin cell was calculated as the ratio of the reversible specific capacity at various temperatures and rates to that at 25 °C and 0.05 C. Similarly, the capacity retention of the LFP||graphite pouch cell was determined by the ratio of the reversible specific capacity at different temperatures and rates to the value at 25 °C and 0.5 C.

The following lists the full names of the reagents mentioned in the table:

LiNi_0.5_Co_0.2_Mn_0.3_O_2_ (NCM523)

LiNi_0.8_Mn_0.1_Co_0.1_O_2_ (NCM811)

Trans-4,5-di-fluoroethylene carbonate (DiFEC)

Methyl (2,2,2-trifluoroethyl) carbonate (MTFC)

Hexafluoroisopropyl methyl ether (HFME)

*N*,*N*-dimethyltrifluoromethane-sulfonamide (TFMSA)

1,1,2,2-tetrafluoroethyl-2,2,3,3-tetrafluoropropyl ether (TTE)

Fluobenzene (FB)

cyclopentyl methyl ether (CPME)

Fluoroethylene carbonate (FEC)

Ethyl trifluoroacetate (ETFA)

**Table S4.** Comparison of the various temperature and rate performance of lithium metal batteries (LMBs) with PDOL-based electrolytes in the literature and the DOL-based electrolytes developed in this study.

| Ref. | Journal | Initiator | Plasticizer/Additive | Electrode system | Temperature (°C) | Rate (C) | Specific capacity (mAh g^−1^) |
| --- | --- | --- | --- | --- | --- | --- | --- |
| [24] | **Sci. Adv.** | LiPF_6_ | DME | LFP\|\|Li | 25 (Room temperature) | 0.5 | ~135 |
|  |  |  |  | NCM622\|\|Li |  | 0.1 | ~165 |
| [25] | **Adv. Funct. Mater.** | LiPF_6_ | DME/PI NF | LFP\|\|Li | 25 | 1 | 96 |
| [26] | **Mater. Today Energy** | LiFSI | DME | LFP\|\|Li | 25 | 5 | 79.2 |
| [27] | **Chem. Eng. J.** | LiDFOB | SN/FEC | LFP\|\|Li | 25 | 6 | 128.3 |
|  |  |  |  | NCM811\|\|Li |  | 1 | 136.8 |
| [28] | **J. Mater. Chem. A** | LiDFOB | SN | LFP\|\|Li | 25 | 5 | 92.6 |
|  |  |  |  | LiMn_2_O_4_\|\|Li |  | 0.5 | 88.4 |
|  |  |  |  | LCO\|\|Li | 40 | 0.1 | 138.3 |
| [29] | **J. Mater. Chem. A** | Mg(OTf)_2_ | FEC | LFP\|\|Li | 25 | 5 | 100 |
| [30] | **Adv. Sci.** | Al(OTf)_3_ | PDA | LFP\|\|Li | 25 | 5 | ~90 |
| [31] | **ACS Appl. Mater. Interfaces** | / | DADS | LFP\|\|Li | -40 | 0.1 | 45 |
| [32] | **Adv. Energy Mater.** | LiPF_6_ | MP/FEC | LFP\|\|Li | -30 | 0.1 | ~50 |
|  |  |  |  |  | 23 | 4 | 113 |
|  |  |  |  | NCM811\|\|Li | -30 | 0.1 | 64 |
|  |  |  |  |  | 23 | 4 | 71 |
| **This work**  **DOL-based** | | / | / | LFP\|\|Li | -40 | 0.5 | 28.3 |
|  |  |  |  |  | 25 | 20 | 106.6 |
|  |  |  |  |  | 60 | 20 | 128.4 |

**Note:**

The following lists the full names of the reagents mentioned in the table:

polyimide nanofiber (PI NF)

Succinonitrile (SN)

polydopamine (PDA)

diallyl disulfide (DADS)

methyl propionate (MP)

**References**

[1] K. Jiang, Q. Li, S. Fan, *Nature* **2002**, *419*, 801.

[2] K. Wang, S. Luo, Y. Wu, X. He, F. Zhao, J. Wang, K. Jiang, S. Fan, *Adv. Funct. Mater.* **2013**, *23*, 846.

[3] R. L. C. Akkermans, N. A. Spenley, S. H. Robertson, *Mol. Simul.* **2021**, *47*, 540.

[4] P. J. Stephen, F. J. Devlin, C. F. Chabalowski, M. J. Frisch, *J. Phys. Chem.* **1994**, *98*, 11623.

[5] R. Krishnan, J. S. Binkley, R. Seeger, J. A. Pople, *J. Chem. Phys.* **1980**, *72*, 650.

[6] T. Lu, F. Chen, *J. Comput. Chem.* **2011**, *33*, 580.

[7] T. Lu, *J. Chem. Phys.* **2024**, *161*, 082503.

[8] J. Zhang, T. Lu, *Phys. Chem. Chem. Phys.* **2021**, *23*, 20323.

[9] L. Cabo-Fernandez, A. R. Neale, F. Braga, I. V. Sazanovich, R. Kostecki, L. J. Hardwick, *Phys. Chem. Chem. Phys.* **2019**, *21*, 23833.

[10] E. R. Logan, E. M. Tonita, K. L. Gering, J. Li, X. Ma, L. Y. Beaulieu, J. R. Dahn, *J. Electrochem. Soc.* **2018**, *165*, A21.

[11] Q. Li, G. Liu, H. Cheng, Q. Sun, J. Zhang, J. Ming, *Chem. Eur. J.* **2021**, *27*, 15842.

[12] Laurence Christian, Jean-François Gal, *Lewis Basicity and Affinity Scales: Data and Measurement*, John Wiley & Sons, **2009**.

[13] K. T. Leffek, P. Pruszynski, K. Thanapaalasingham, *Can. J. Chem.* **1989**, *67*, 590.

[14] K. Kaupmees, A. Trummal, I. Leito, *Croat. Chem. Acta* **2014**, *87*, 385.

[15] T. Rodima, I. Kaljurand, A. Pihl, V. Ma, *J. Org. Chem.* **2002**, *67*, 1873.

[16] T. FUJII, H. NISHIDA, Y. ABIRU, M. YAMAMOTO, M. KISE, *Chem. Pharm. Bull.* **1995**, *43*, 1872.

[17] X. Zheng, Z. Cao, W. Luo, S. Weng, X. Zhang, D. Wang, Z. Zhu, H. Du, X. Wang, L. Qie, H. Zheng, Y. Huang, *Adv. Mater.* **2023**, *35*, 2210115.

[18] A. Wang, Y. Song, Z. Zhao, X. Li, Z. Hu, J. Luo, *Adv. Funct. Mater.* **2023**, *33*, 2302503.

[19] M. Qin, M. Liu, Z. Zeng, Q. Wu, Y. Wu, H. Zhang, S. Lei, S. Cheng, J. Xie, *Adv. Energy Mater.* **2022**, *12*, 2201801.

[20] B. Nan, L. Chen, N. D. Rodrigo, O. Borodin, N. Piao, J. Xia, T. Pollard, S. Hou, J. Zhang, X. Ji, J. Xu, X. Zhang, L. Ma, X. He, S. Liu, H. Wan, E. Hu, W. Zhang, K. Xu, X. Q. Yang, B. Lucht, C. Wang, *Angew. Chemie - Int. Ed.* **2022**, *61*, e202205967.

[21] J. Xu, J. Zhang, T. P. Pollard, Q. Li, S. Tan, S. Hou, H. Wan, F. Chen, H. He, E. Hu, K. Xu, X.-Q. Yang, O. Borodin, C. Wang, *Nature* **2023**, *614*, 694.

[22] Z. Wang, R. Han, D. Huang, Y. Wei, H. Song, Y. Liu, J. Xue, H. Zhang, F. Zhang, L. Liu, S. Weng, S. Lu, J. Xu, X. Wu, Z. Wei, *ACS Nano* **2023**, *17*, 18103.

[23] Y. Yang, Z. Fang, Y. Yin, Y. Cao, Y. Wang, X. Dong, Y. Xia, *Angew. Chemie* **2022**, *134*, e202208345.

[24] F. Liu, W. Wang, Y. Yin, S. Zhang, J. Shi, L. Wang, *Sci. Adv.* **2018**, *4*, eaat5383.

[25] Y. Huang, S. Liu, Q. Chen, K. Jiao, B. Ding, J. Yan, *Adv. Funct. Mater.* **2022**, *32*, 2201496.

[26] H. Cheng, J. Zhu, H. Jin, C. Gao, H. Liu, N. Cai, Y. Liu, P. Zhang, M. Wang, *Mater. Today Energy* **2021**, *20*, 100623.

[27] Y. Liu, Y. Xu, *Chem. Eng. J.* **2022**, *433*, 134471.

[28] Q. Liu, B. Cai, S. Li, Q. Yu, F. Lv, F. Kang, Q. Wang, B. Li, *J. Mater. Chem. A* **2020**, *8*, 7197.

[29] Z. Li, W. Tang, Y. Deng, M. Zhou, X. Wang, R. Liu, C. Wang, *J. Mater. Chem. A* **2022**, *10*, 23047.

[30] D. Chen, M. Zhu, P. Kang, T. Zhu, H. Yuan, J. Lan, X. Yang, *Adv. Sci.* **2022**, *9*, 2103663.

[31] J. Zhou, H. Ji, Y. Qian, J. Liu, T. Yan, C. Yan, T. Qian, *ACS Appl. Mater. Interfaces* **2021**, *13*, 48810.

[32] J. Yu, X. Lin, J. Liu, J. T. T. Yu, M. J. Robson, G. Zhou, H. M. Law, H. Wang, B. Z. Tang, F. Ciucci, *Adv. Energy Mater.* **2022**, *12*, 2102932.
